# Natural variation in the regulation of neurodevelopmental genes modifies flight performance in *Drosophila*

**DOI:** 10.1371/journal.pgen.1008887

**Published:** 2021-03-18

**Authors:** Adam N. Spierer, Jim A. Mossman, Samuel Pattillo Smith, Lorin Crawford, Sohini Ramachandran, David M. Rand

**Affiliations:** 1 Department of Ecology and Evolutionary Biology, Brown University, Providence, Rhode Island, United States of America; 2 Center for Computational Molecular Biology, Brown University, Providence, Rhode Island, United States of America; 3 Microsoft Research New England, Cambridge, Massachusetts, United States of America; UNITED STATES

## Abstract

The winged insects of the order *Diptera* are colloquially named for their most recognizable phenotype: flight. These insects rely on flight for a number of important life history traits, such as dispersal, foraging, and courtship. Despite the importance of flight, relatively little is known about the genetic architecture of flight performance. Accordingly, we sought to uncover the genetic modifiers of flight using a measure of flies’ reaction and response to an abrupt drop in a vertical flight column. We conducted a genome wide association study (GWAS) using 197 of the *Drosophila* Genetic Reference Panel (DGRP) lines, and identified a combination of additive and marginal variants, epistatic interactions, whole genes, and enrichment across interaction networks. *Egfr*, a highly pleiotropic developmental gene, was among the most significant additive variants identified. We functionally validated 13 of the additive candidate genes’ (*Adgf-A/Adgf-A2/CG32181*, *bru1*, *CadN*, *flapper* (*CG11073*), *CG15236*, *flippy* (*CG9766*), *CREG*, *Dscam4*, *form3*, *fry*, *Lasp/CG9692*, *Pde6*, *Snoo*), and introduce a novel approach to whole gene significance screens: PEGASUS_flies. Additionally, we identified *ppk23*, an Acid Sensing Ion Channel (ASIC) homolog, as an important hub for epistatic interactions. We propose a model that suggests genetic modifiers of wing and muscle morphology, nervous system development and function, BMP signaling, sexually dimorphic neural wiring, and gene regulation are all important for the observed differences flight performance in a natural population. Additionally, these results represent a snapshot of the genetic modifiers affecting drop-response flight performance in *Drosophila*, with implications for other insects.

## Author summary

Insect flight is a widely recognizable phenotype of many winged insects, hence the name: flies. While fruit flies, or *Drosophila melanogaster*, are a genetically tractable model, flight performance is a highly integrative phenotype, and therefore challenging to identify comprehensively which genetic modifiers contribute to its genetic architecture. Accordingly, we screened 197 Drosophila Genetic Reference Panel lines for their ability to react and respond to an abrupt drop. Using several computational approaches, we identified additive, marginal, and epistatic variants, as well as whole genes and altered sub-networks of gene-gene and protein-protein interaction networks that contribute to variation in flight performance. More generally, we demonstrate the benefits of employing multiple methodologies to elucidate the genetic architecture of complex traits. Many variants and genes mapped to regions of the genome that affect neurodevelopment, wing and muscle development, and regulation of gene expression. We also introduce PEGASUS_flies, a *Drosophila*-adapted version of the PEGASUS platform first used in human studies, to infer gene-level significance of association based on the gene’s distribution of individual variant *P*-values. Our results contribute to the debate over the relative importance of individual, additive factors and epistatic, or higher order, interactions, in the mapping of genotype to phenotype.

## Introduction

Flight is one of the most distinguishing features of many winged insects, especially the taxonomic order *Diptera*. Colloquially named “flies,” these insects rely on their namesake for many facets of their life history: dispersal, foraging, evasion, migration, and mate finding [1]. Because flight is central to the life history of flies, many of the genes essential for flight are strongly conserved [2, 3]. Flight is an epiphenomenon, comprised of many highly integrated systems working together. As such, flight performance is likely to be a continuously varying trait with a complex genetic basis.

Evaluating the genetic architecture of complex traits is inherently challenging due to the fact that many genes each contribute a small fraction to the overall trait variation [4, 5]. The omnigenic model [6, 7] frames the genetic architecture of complex traits as a network of all expressed genes where variation stems from the action of a very large number of peripheral modifiers that alter the action of core genes and pathways at the center of the network. Genes in the network are connected by edges representing various interaction types (gene-gene, protein-protein, epistatic, etc.). In the case of flight performance, for example, central genes like *Wingless* [8] and *Act88F* [9] are essential for wing and indirect flight muscle development, respectively, while peripheral genes would have more subtle effects on flight from systems like metabolism [10], muscle function [11], neuronal function [12, 13], and anatomical development [14, 15]. Peripheral genes are less likely to experience the same degree of purifying selection as central genes, meaning they are more likely to harbor natural variants that can have subtle effects on phenotype.

We can leverage the diversity of natural variants in a population to uncover novel associations between genotype and phenotype, via Genome Wide Association Study (GWAS). The *Drosophila* Genetics Reference Panel (DGRP) is a panel of 205 inbred and genetically distinct *Drosophila melanogaster* lines representing a snapshot of natural variation in a population [16, 17]. Previous studies on complex and highly polygenic, quantitative traits have identified many candidate loci contributing to insect- and *Drosophila*-specific traits [18–20], as well as traits affecting human health and disease [21–24].

We designed this study to identify genetic modifiers of flight performance and map out a possible network of the underlying genetic architecture. We screened males and females from 197 DGRP lines, then analyzed both sexes, their average, and their difference. We took a multifaceted approach, identifying modifiers at the individual variant (n_additive_ = 180 variants; n_marginal_ = 70 variants; n_epistatic_ = 12,161 variants) and network levels (n = 539 genes). We developed a novel application of the human-based PEGASUS program [25] for use with *Drosophila* and DGRP studies that identified 72 whole genes of significance: PEGASUS_flies. In addition to these findings, we successfully validated 13 candidate genes from the additive approach using *Mi{ET1}* mutational insertions.

Taken together, our results strongly suggest variation in flight performance across natural populations is affected by non-coding regions of the genome. Many of the genes affecting the genetic architecture for flight performance are known to affect 1) neural development, 2) development of flight musculature, 3) development of wing morphology, and 4) regulation of gene expression. Based on the validation of our candidate genes and strongly significant genes from different analyses, we propose a model to summarize how these genetic modifiers work to affect flight performance.

## Results

### Variation in flight performance across the DGRP

**Fig 1:**
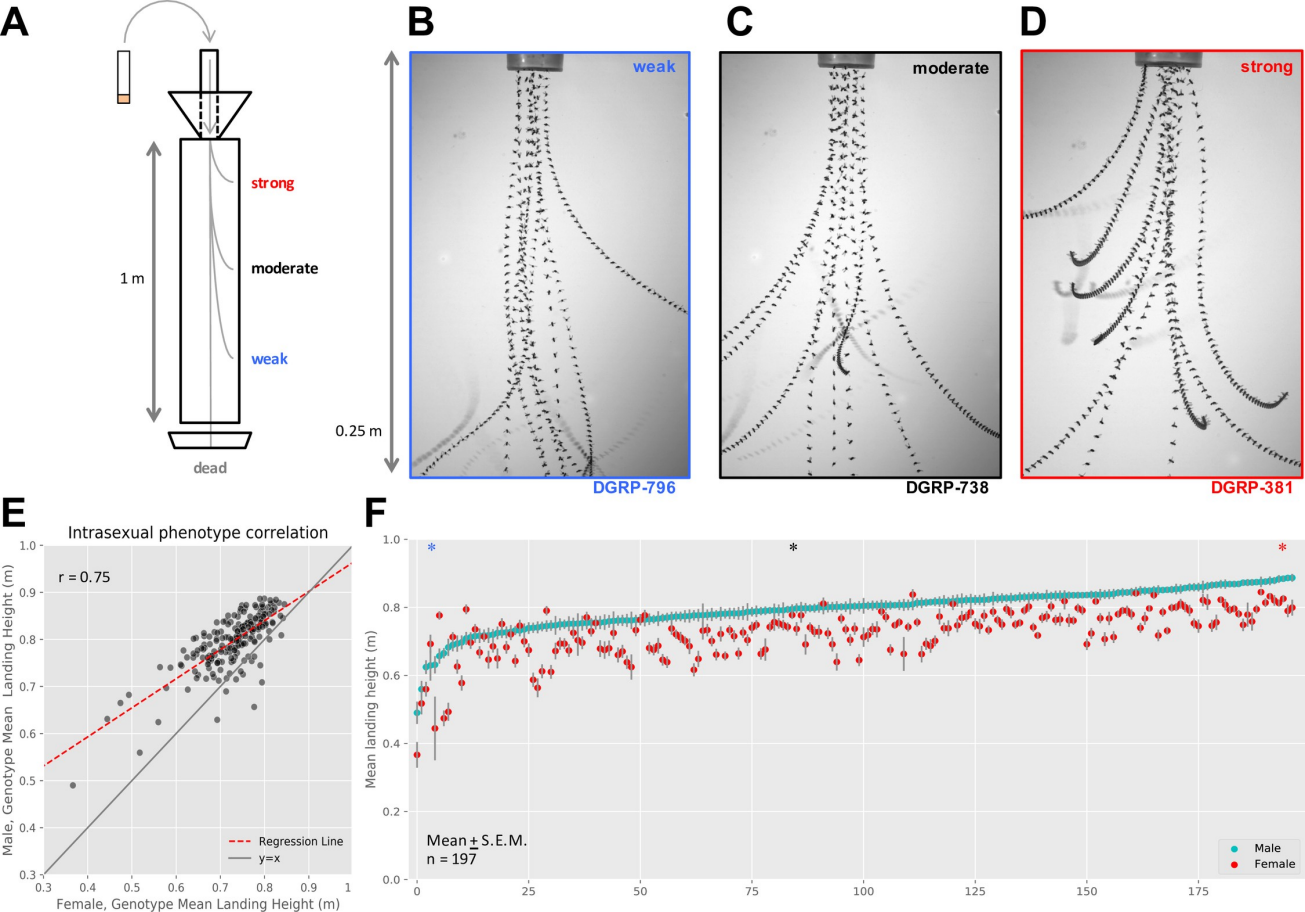
DGRP lines show differences in flight performance across lines. (A) The flight performance assay measures the average landing height of flies as they fall through a flight column. Vials of flies are sent down the top chute and abruptly stop at the bottom, ejecting flies into a meter-long column. Falling flies will instinctively right themselves and fly to the periphery, doing so at different times (and therefore landing at different heights) depending on their performance ability. (B-D) Collapsed z-stacks of every 10^th^ frame from a high-speed video recorded from the top quarter (0.25m) of the flight column illustrate these performance differences in (B) weak, (C) intermediate, and (D) strong genotypes. (E) Sexual dimorphism exists within genotypes (deviation of red dashed regression line from y = x solid gray line), though sexes are well correlated (r = 0.75, n = 197, *P* < 1e-36). (F) Sexually dimorphic performances are also apparent in the distribution of mean landing heights for each male (cyan) and female (red) genotype pair (mean ± S.E.M.). Sex-genotype pairs are sorted in order of increasing male mean landing height. Performances for genotypes in B-D are indicated on the distribution with the corresponding color-coded asterisk (*) above the respective genotype position.

Cohorts of approximately 80 flies from 197 lines of the DGRP (S1 Table) were tested for flight performance using a flight column [26] (Fig 1A). We recorded high-speed videos for a weak, intermediate, and strong genotype entering the flight column (Fig 1B–1D and File 1 in https://doi.org/10.7910/DVN/ZTDHDC). Based on these videos, we concluded this assay is best for measuring the reaction and response to an abrupt drop.

There was strong agreement between the mean landing height of males and females for each genotype (r = 0.75; Fig 1E), with males showing higher landing heights than females (male: 0.80m ± 0.06 SD; female: 0.73m ± 0.07 SD; Fig 1F and S2 Table). Since landing height was somewhat sexually dimorphic, we calculated broad sense heritability (*H*^*2*^) separately for each sex (*H*^*2*^_*Male*_ = 13.5%; *H*^*2*^_*Female*_ = 14.4%), and confirmed the assay’s repeatability by retesting 12 lines of varied ability, reared 10 generations apart (r = 0.95; S1 Fig). Together, these results point toward genetic (rather than experimental or environmental) variation as a main source of variation in flight between the individual DGRP lines. In addition to analyzing males and females, we also analyzed the average (sex-average) and difference (sex-difference) between sexes (S2 Fig).

Before running the association analysis, we tested whether flight performance was a unique phenotype. We compared our phenotype scores for males and female against publicly available phenotypes on the DGRP2 webserver, as well as visual senescence at three time points [22]. We found no significant regression between flight performance and any of the phenotypes in either sex after correcting for multiple testing (*P* ≤ 1.67e-3; S3 Table). This result suggests our measure of flight performance is a unique phenotype among those reported.

### Association of additive SNPs with flight performance

**Fig 2:**
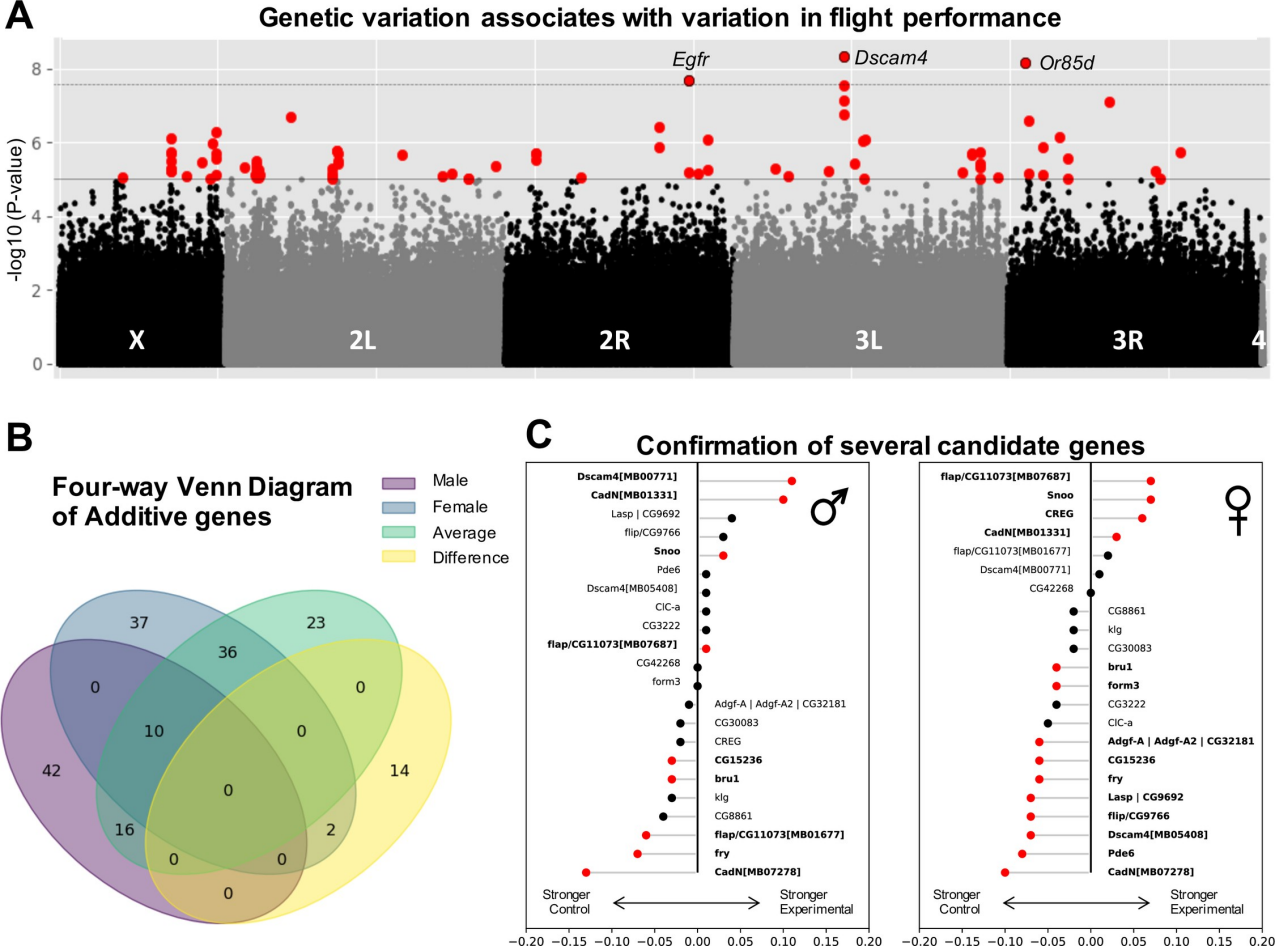
Variation in flight performance associated with several additive variants, some of which were functionally validated. (A) An additive screen for genetic variants identified several variants that exceeded the traditional [29] DGRP (*P* ≤ 1e-5) threshold (gray line). Significant variants (red) were spread throughout the genome on all but chromosome 4. Variants for the sex-averaged phenotype are pictured, though other sex-based phenotypes had similar profiles (S5 Fig). (B) Approximately half of all variants were shared with at least one other sex-based analysis, while the other half of all variants was exclusive to a single analysis. (C) Candidate genes were selected from the Bonferroni-corrected variants and those most significant in the sex-average analysis, for which transgenic flies were publicly available. Both sexes were tested for flight performance. Validated genes were determined if there was a significant difference between experimental lines homozygous for an insertional mutant in the candidate gene and their background control lines lacking the insertional mutant (red points, Mann-Whitney-U test, *P* ≤ 0.05). Very significant candidate genes (*CadN*, *flapper* (*CG11073*), and *Dscam4*) each had two independent validation lines.

We conducted a Genome Wide Association Study (GWAS) to identify genetic markers associated with flight performance. We performed an analysis with 1,901,174 common variants (MAF ≥ 0.05) on the additive genetic effects of four sex-based phenotypes: males, females, sex-average, and sex-difference. Some phenotypes covaried with the presence of major inversions (S4 Table), so we analyzed association results using a mixed model (Fig 2A) to account for *Wolbachia* infection status, presence of inversions, and polygenic relatedness (S3 and S4 Figs), calculated using the DGRP2 webserver.

We filtered additive variants with a strict Bonferroni threshold (*P* ≤ 2.63e-8). Taking a minSNP approach to identify significant genes if their lowest (most significant) variant *P*-value crossed a threshold [25], we identified six unique variants. Five of these variants mapped to six genes (*CG15236*, *CG34215*, *Dscam4*, *Egfr*, *fd96Ca*, *Or85d*) (Table 1). Variants mapping to *Egfr* and *fd96Ca* also mapped to cis-regulatory elements (transcription factor binding sites (TFBS) and a silencer) known to activate during embryogenesis [27, 28]. Of note, *Dscam4* was deemed “damaged” in 38 of the lines tested [17]; however, the difference between mean landing heights of flies with the damaged vs. undamaged allele was less than 1 cm (*P* = 0.32, Welch’s T-test). The allele causing the damaged condition is not the allele associated with flight performance.

**Table 1:** Six additive variants surpassed the Bonferroni significance threshold.

Variant	MAF	Annotation		
		Gene (Dmel)	Gene (Hsap)	Regulatory Region
2R_17433667_SNP	0.05128	*Egfr* (intron)	EGFR	TFBS (*bcd*, *da*, *dl*, *gt*, *hb*, *kni*, *Med*, *prd*, *sna*, *tll*, *twi*, *disco*, *Trl*)
2R_2718036_DEL	0.05641	*CG15236* (intron) *CG34215* (downstream, 764 bp)	- -	-
3L_8237821_SNP	0.0829	*Dscam4* (intron)	DSCAM	-
3R_20907854_SNP	0.06557	*fd96Ca* (upstream, 552bp)	FOXB1/FOXB2	TFBS (*dl*) Silencer (*HDAC*)
3R_4379159_SNP	0.05263	*Or85d* (non-synonymous, C277Y)	-	-
3R_9684126_SNP	0.1514	-	-	-

These variants represented all four sex-based phenotypes and were typically near the minor allele frequency (MAF) ≥ 0.05 limit. All but one variant mapped to a gene in *Drosophila* (Dmel), and three had human orthologs (Hsap). Additionally, two SNPs mapped to embryonic transcription factor binding sites (TFBS) and a silencer region.

Using the traditional DGRP significance threshold (*P* ≤ 1e-5) [29], we identified 180 variants across all four sex-based phenotypes (Figs 2B and S5 and S5 Tables). All of the identified variants passed their permutation test (n = 10,000) significance thresholds for each sex-based phenotype, suggesting variants were not associated with flight by random chance. The individual additive variant with the largest effect size mapped to an intron in *epidermal growth factor receptor* (*Egfr*; human homolog *EGFR*) and contributed a 4.5 cm difference in landing height (or 0.97% of the sum of all significant variants) for males and 6.4 cm (1.1% of the sum of all significant variants) for females. For reference, the variant with the smallest significant effect size was 0.017 cm (or 0.0036% of the sum of all significant variants) for males and 0.57 cm (or 0.095% of the sum of all significant variants) for females. Notably, *Egfr* encodes a key transmembrane tyrosine kinase receptor and was previously identified as a locus influencing wing shape in the DGRP [30, 31]. As a pleiotropic gene, *Egfr* affects developmental and homeostatic processes throughout the life and anatomy of the fly. It is known for its role in embryonic patterning and has implications in tumorigenesis [32, 33]. When cis-regulatory elements lie in important developmental genes, their effects can magnify as the organism continues through development. These effects can magnify further for genes like *Egfr* that are receptors known to affect other developmental processes [31]. Accordingly, this variant in *Egfr* mapped to several overlapping transcription factor binding sites (TFBS) for transcription factors known to affect embryonic development in a highly dose-dependent manner (*bcd*, *da*, *dl*, *gt*, *hb*, *kni*, *Med*, *prd*, *sna*, *tll*, *twi*, *disco*, *Trl*) [34–37], suggesting this variant may play a similar role.

Across the four sex-based analyses, all but 19 variants mapped to intergenic or non-coding regions (putative cis-regulatory regions). Of the non-coding variants, 149 mapped to 136 unique genes (Table 2). These included development and function of the nervous system and neuromuscular junctions, muscle, cuticle and wing morphogenesis, endoplasmic reticulum and Golgi body functions, and regulation of translation. Approximately half of all variants were present in two or three sex-based analyses, while the remainder were unique to one (Fig 2B). Several variants mapped to transcription factors broadly affecting development and neurogenesis [38, 39]. Despite the enrichment for several annotations, we failed to identify any significant gene ontology (GO) categories using GOwinda [40], a GWAS-specific gene set enrichment analysis.

**Table 2:** Aggregated gene and variant counts by sex-based phenotype for each analysis.

**Additive analysis**				
	**Male**	**Female**	**Sex-Average**	**Sex-Different**
Bonferroni variants (*P* ≤ 2.63e-8)	1	4	3	1
Bonferroni minSNP genes (*P* ≤ 2.63e-8)	1	4	3	2
Conventional variants (*P* ≤ 1.00e-5)	68	85	85	16
Conventional minSNP genes (*P* ≤ 1e-5)	56	73	69	11
**Marginal analysis**				
	**Male**	**Female**	**Sex-Average**	**Sex-Different**
Bonferroni Variants (*P* ≤ 2.56e-8)	7	13	62	0
minSNP Genes (P ≤ 2.56e-8)	5	7	21	0
**Epistatic analysis**				
	**Male** (*P* ≤ 3.75e-9)	**Female** (*P* ≤ 2.02e-9)	**Sex-Average** (*P* ≤ 4.24e-10)	**Sex-Different**
Paired Primary Variants	1	5	18	0
Paired Primary Genes	1	2	6	0
Paired Secondary Variants	42	2188	6139	0
Paired Secondary Genes	28	1061	2419	0
**Whole gene analysis**				
	**Male**	**Female**	**Sex-Average**	**Sex-Different**
Bonferroni (*P* ≤ 3.01e-6)	23	29	25	23
**Network analysis**				
	**All sex-based phenotypes**			
Sub-Networks	9			

Each analysis identified different genetic modifiers (variants, genes, networks). For each analysis, the different variant-, gene-, and network-based analyses identified separate genetic features associated with flight performance.

### General development and neurodevelopmental genes validated to affect flight performance

We performed functional validations on a subset of the genes mapped from variants identified in the Bonferroni and sex-average analysis. We identified 21 unique candidate genes for which a *Minos* enhancer trap *Mi{ET1}* insertional mutation line [41] was publicly available [42] (S1 Table; *Adgf-A/Adgf-A2/CG32181*, *bru1*, *CadN*, *CG11073*, *CG15236*, *CG9766*, *CREG*, *Dscam4*, *form3*, *fry*, *Lasp/CG9692*, *Pde6*, *Snoo*). Three additional stocks for *CadN*, *Dscam4*, and *CG11073* were also tested for their strength of association. *CREG* was also included as a negative control, since no variant exceeded the 25^th^ percentile for significance (*P* = 0.25).

Candidate genes were functionally validated by comparing the distribution in mean landing heights of stocks homozygous for the insertion and their paired control counterpart (S6 Fig) using a Mann-Whitney-U test (Fig 2C and S6 Table). Several candidate genes were involved in development of the nervous system (*CadN*, *CG9766*, *CG11073*, *CG15236*, *Dscam4*, *fry*, and *Snoo*) [8, 43–51], muscle development (*bru1* and *Lasp*) [11, 52, 53], and transcriptional regulation of gene expression (*CREG*) [54] Following successful validation of *CG9766* and *CG11073*, two unnamed candidate genes, we are naming them *flippy* (*flip*) and *flapper* (*flap*) based on the flipping and flapping motions of weaker flies struggling to right themselves in the flight performance assay. For more information on these two genes, see S1 Text for "Putative roles for *flippy* and *flapper*".

### Association of gene-level significance and interaction networks with flight performance

The minSNP approach on the additive variants prioritizes the identification of genes containing variants with larger effects [25]. However, this approach ignores linkage blocks and gene length, which can bias results. It is important to account for gene length because some genes can be long and exceed 100kb (e.g. neurodevelopmental genes such as *CadN*, 131kb). One alternative approach is Precise, Efficient Gene Association Score Using SNPs (PEGASUS), which assesses whole gene significance scores by comparing a gene’s variant *P*-value distributions against a null chi-squared distribution [25]. This approach enriches for whole genes of moderate effect and enables the identification of genes that might go undetected in a minSNP approach.

**Fig 3:**
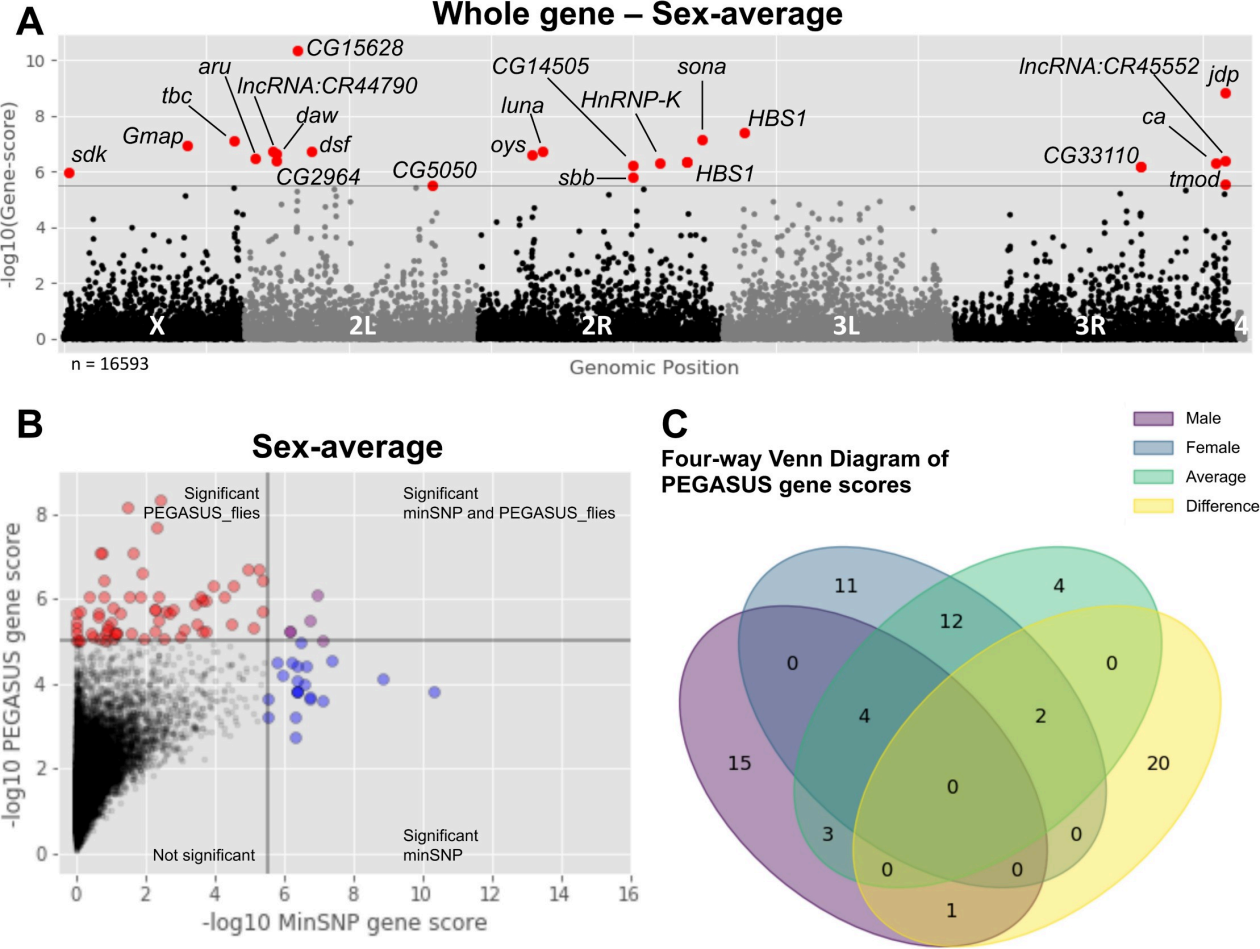
PEGASUS_flies identifies different genetic modifiers than the additive screen. (A) PEGASUS_flies results plotted as a Manhattan plot. For the sex-average phenotype, several genes (red points, labeled with gene symbol) exceeded a strict Bonferroni significance threshold (gray dashed line, *P* ≤ 3.43e-6) identified several genes. (B) PEGASUS_flies prioritizes genetic modifiers of moderate effect, taking into account linkage blocks and gene length. Significant PEGASUS_flies (red) compared against genes significant under a minSNP approach for additive variants (blue) have very little overlap between the two sets (purple). (C) Many of the genes PEGASUS_flies identified were unique to a sex-based phenotype, though the sex-average genes were generally found in other analyses.

Because PEGASUS is configured for human populations, we developed PEGASUS_flies, a modified version for *Drosophila* <https://github.com/ramachandran-lab/PEGASUS_flies>. This platform is configured to work with DGRP data sets, and can be customized to accept other *Drosophila*/model screening panels. From our additive variants, PEGASUS_flies identified 72 unique genes across all sex-based phenotypes whose gene scores passed a Bonferroni threshold (*P* ≤ 3.03e-6; Fig 3A and S7 Table). The significant genes were generally different from those identified in the additive minSNP analyses (Figs 3B and S7), though 15 were shared (*aru*, *bves*, *CG17839*, *CG32506*, *CG33110*, *fry*, *Gmap*, *Mbs*, *mip40*, *mxt*, *oys*, *Pdp1*, *Rab30*, *sdk*, *VAChT*). The relatively low overlap between these two gene sets is to be expected, since they prioritize variants of large effect (minSNP) vs. whole genes of moderate effect (PEGASUS_flies). Overall, gene annotations were enriched for neural development and function, wing and general development, Rab GTPase activity, and regulators of transcription. Different sex-based phenotypes varied in how unique certain whole genes were to a given phenotype (Fig 3C). Genes identified in the sex-average analysis were generally shared with the male and female phenotypes, while genes in the sex-difference analysis were typically unique. Interestingly, *dsf* and *sdk* were both present in the sex-average and sex-difference, and *Ccn* was present in both the male and sex-difference. *Ccn* was also located in the Missouri insertion of chromosome 3R (In_3R_Mo; a significant covariate for male and sex-average analyses), though PEGASUS_flies accounts for linkage blocks so *Ccn* is still significant.

Taking advantage of the gene-level significance scores, we leveraged publicly available gene-gene and protein-protein interaction networks to identify altered sub-networks of genes that connect to the flight performance phenotype. A local False Discovery Rate (lFDR) was calculated for each sex-based phenotype (S8 Table), for which gene-scores were either–log10 transformed if they passed, or set to 0 if they did not. Transformed scores for each sex-based phenotype were analyzed together in Hierarchical HotNet [55], which returned a consensus network consisting of nine sub-networks of genes (S9 Table). The largest network identified 512 genes and was significantly enriched for several GO terms, including transcription factor binding, histone and chromatin modification, regulation of nervous system development, and regulation of apoptosis (S10 Table). The other eight networks were comprised of 27 genes, which together had several significant GO terms, including regulation of gene expression through alternative splicing, maintenance of the intestinal epithelium, and the Atg1/ULK1 kinase complex (S11 Table).

### Association of epistatic interactions with flight performance

**Fig 4:**
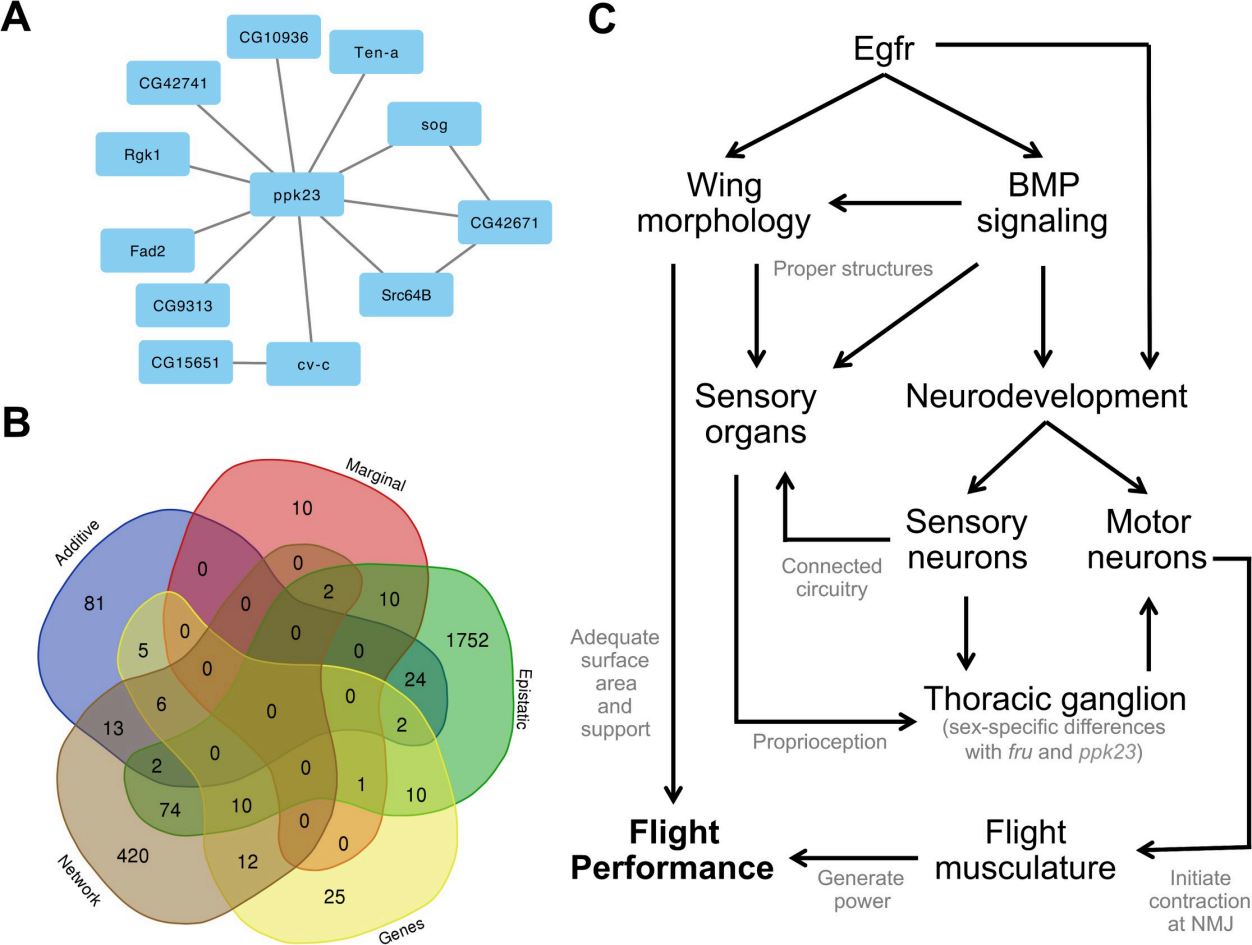
Flight performance is a larger complex trait comprised of several smaller traits. (A) The genetic architecture of epistatically interacting genes shared *ppk23* as a more central node. (B) Whole genes and minSNP genes were not identified in more than three analyses, while roughly half or more genes were unique to each analysis. (C) Flight performance has a complex genetic architecture, with the key developmental gene *Egfr* and BMP signaling pathway contributing to wing and neurodevelopment. These processes are both important for structuring the sensory organs that enable the fly to use mechanosensory channels for proprioception. Signals from the sensory organs on the wing, head, and body travel to the brain and thoracic ganglion, which sends signals through the motor neurons to the direct and indirect flight musculature that is also differentially assembled and innervated to generate power and control the wing angle during flight.

Epistatic interactions account for a substantial fraction of genetic variation in complex traits [56], but they are computationally and statistically challenging to identify. To circumvent the barriers associated with an exhaustive, pairwise search (n = 1.81E12), we focused our search area with MArginal ePIstasis Test (MAPIT). MAPIT is a linear mixed modeling approach that identifies variants more likely to have an effect on other variants. These putative hub variants represent more central and interconnected genes in a larger genetic network proposed by the omnigenic model [6, 7]. Accordingly, we identified 70 unique, significant marginal variants exceeding a Bonferroni threshold (*P* ≤ 2.56e-8) across male, female, and sex-average phenotypes, but none in the sex-difference analysis (S8 Fig and S12 Table). We tested these 70 marginal variants for pairwise interactions against all other SNPs in the data set and found 20 unique marginal variants with significant pairwise interactions that passed the Bonferroni threshold (S13 Table). Some of these interactions were between genes containing marginal variants, but not necessarily between the marginal variants themselves (Fig 4A). For example, *ppk23* and *sog* both contain significant marginal variants but the significant pairwise interactions were between marginal and non-marginal variants. This highlights an important benefit of using several approaches for finding different types of additive, marginal, or epistatic effects within the same gene. Additionally, since marginal variants represent those more likely to interact with other variants, their interaction between genes containing significant marginal variants suggests a highly interconnected genetic architecture underlying flight performance. The breadth of epistatic interactions from a small, focused subset of marginal variants supports an important role for epistasis in the genetic architecture of flight performance. There are likely many more variants that interact with one another beyond the limited subset of 70 marginal variants we tested. We briefly recapitulate these findings in order of the male, female, and sex-average results, though a more comprehensive survey is available in S1 Text under "Association of epistatic interactions with flight performance, continued".

In males, there were seven significant marginal variants that mapped to five genes (*CG5645*, *CG18507*, *cv-c*, *sog*, *Ten-a*). Of the variants, only the one (X_15527230_SNP) that mapped to a novel transcription start site in the BMP antagonist of *short gastrulation* (*sog*; human ortholog of *CHRD*) had significant interactions. This marginal SNP interacted with 42 other variants across 28 unique genes (S13 Table). A quarter of these genes are important in neuron development, signaling, and function.

In females, there were 14 significant marginal variants that mapped to several intergenic variants and six genes (*CG6123*, *CG7573*, *CG42741*, *ppk23*, *Src64B*, *twi*). Most notably, four variants mapped to a 1,002 bp region downstream of *pickpocket 23* (*ppk23*; human homologs in ASIC gene family) and accounted for the majority of epistatic interactions in the female analysis. *ppk23* is a member of the degenerin (DEG)/epithelial Na+ channel (ENaC) gene family that functions as subunits of non-voltage gated, amiloride-sensitive cation channels. It is involved in chemo- and mechanosensation, typically in the context of foraging, pheromone detection, and courtship behaviors [57, 58].

In the sex-average analysis, there were 62 significant marginal variants that mapped to intergenic regions and 21 genes (*Art2*, *CG10936*, *CG15630*, *CG15651*, *CG18507*, *CG3921*, *CG42671*, *CG42741*, *CG5645*, *CG6123*, *CG9313*, *CR44176*, *cv-c*, *Fad2*, *natalisin*, *ppk23*, *Rbfox1*, *Rgk1*, *Src64B*, *twi*; Fig 4A). Of the 62 marginal variants, 18 had significant epistatic interactions with other variants in the genome, with the majority in intergenic regions (n = 9) and *ppk23* (n = 7), while individual variants mapped to *CG42671*, *CG10936*, *CG9313*, and *CG15651* (S13 Table). Again, *ppk23* had the greatest number of epistatic interactions of a single gene, and many of these interactions were with variants that mapped to genes with significant marginal variants themselves (*A2bp1*, *cv-c*, *Fad2*, *CG9313*, *CG10936*, *CG42741*, *Rgk1*, *sog*, *Src64B*, *twi*, *Ten-a*). Notably, *ppk23*’s mapped interactions were with genes that collectively had significant GO term enrichment for neuronal growth, organization and differentiation (S14 Table). There were two other groups of variants: one group in *CG42671* and the other containing six intergenic variants in a 669 bp region (chr3L:6890373–6891042), that also had an abundance of epistatic interactions with significant enrichment for several GO categories related to development and function of the nervous system (S15 and S16 Tables).

### No evidence for adult transcriptome variation affecting flight performance

Since many variants mapped to cis- and trans-regulatory genes, we sought to test whether regulatory variation was affecting developmental or adult homeostasis. Accordingly, we performed a Weighted Gene Co-expression Network Analysis (WGCNA) [59] using 177 publicly available DGRP transcriptomic profiles for young adults of both sexes [60]. We clustered genes by similarity in expression profile, then correlated those clusters’ eigenvalues with the mean and standard deviation of flight performance, as well as the proportion of flies that fell through the column versus the total assayed. No clusters across sex or phenotype had a significant correlation. This result supports our previous observation that many of the significant variants map to genes involved in pre-adult development, rather than genes that are likely to have variable expression levels as adults assayed under presumably homeostatic conditions [60] (S9 Fig). Accordingly, we recommend future studies on complex traits should explore gene expression or similar phenotypes targeted at relevant developmental stages, rather than only during later or adult stages when a phenotype is measured.

### Flight performance is modulated by an interconnected genetic architecture

The genetic architecture of flight performance is comprised of many different types of genetic modifiers. Many of the variants map to genes that are found across analytic platforms (Fig 4B). Most variants were unique to a single analysis, suggesting that association studies should consider using multiple different analyses to enhance the power to detect variants and genes in their study. However, many genes were identified in two (148) or three (23) analyses. Those involved in three analyses include: *aru*, *CG2964*, *CG13506*, *CG15651*, *CG17839*, *CG42671*, *CycE*, *daw*, *Diap1*, *Egfr*, *fz2*, *Gart*, *Gmap*, *Mbs*, *MED23*, *mip40*, *mxt*, *Pdp1*, *Rab30*, *rhea*, *sog*, *sona*, *Tgi*. This suggests that individual genes can contain variants with different types of effects or have differential contributions to the overall genetic architecture. A complete lookup table of all genes and genes identified from variants is available in S17 Table.

## Discussion

We tested flight performance of 197 DGRP lines, identifying several additive and marginal variants, epistatic interactions, whole genes, and a consensus network of altered sub-networks that associated with variation in our phenotype. Many putative cis-regulatory variants mapped to genes with annotations for wing morphology, indirect flight muscle performance, and development of sensory neurons and neuromuscular junctions. We demonstrate that implementation of complementary approaches can expand the breadth of genetic modifiers identified and improve genomic predictions in mapping the genotype-phenotype landscape [29]. Our study corroborates this observation and lays out four additional computational approaches that can expand on the traditional minSNP output from the DGRP2 webserver. These results expand our understanding of the genetic architecture of complex traits, since they provide greater context at the whole gene (PEGASUS_flies) and interaction/network levels (MAPIT, Hierarchical HotNet, PLINK’s–epistasis feature). Associating genes from individual variants can be tricky [25], and finding epistatic interactors can be computationally and statistically demanding [61]. Furthermore, by combining these approaches, we add to a growing body of literature emphasizing the importance of a multi-faceted approach in elucidating the genetic architecture of complex traits.

### Neurodevelopmental genes play an important role in modifying flight performance

There were a number of genes associated with the development of the nervous system across all analyses, including a notable overlap between the additive minSNP and whole gene screens (*aru*, *ChAT*, *Ccn*, *DIP-δ*, *dsf*, *dsx*, *fry*, *Mbs*, *sdk*, *VAChT*). Successful validation of known genes involved in neurodevelopment (*CadN*, *Dscam4*, *fry*, *Snoo*), confirmed its importance. Specifically, these four genes are known to work together for developing and patterning the small, sensory, hair-like structures that line the fly’s body and wings (microchaete). These four genes are also known to facilitate innervation of these structures and connect them to the central nervous system (CNS) through type IV dendritic arborization sensory neurons [48, 62–66]. Interestingly, we identified 41 pickpocket, olfactory receptor neuron (ORN), gustatory receptor neuron (GRN), and ionotropic receptor (IR) genes that aid in signal reception on microchaete. These important mechano- and chemosensory structures suggest that they might play an important role in flight performance. Of note, only six of these 41 genes were previously identified from an olfactory screen with 14 separate odors in a past DGRP GWA study (*Gr59d*, *Ir41a*, *Ir60d*, *Or24a*, *ppk10* and *ppk12*) [18], suggesting a putative role for these genes in flight performance and a potential explanation for the identification of *Or85d* as a Bonferroni-corrected additive variant in the minSNP analysis.

Interestingly, *pickpocket 23* (*ppk23*) was identified as a central node in the marginal and epistasis analyses. Pickpocket family genes are a conserved group of acid sensing ion channels (ASIC family in humans) that are known to work with chemosensory receptors for pheromone detection and are often studied in the context of courtship [57, 67], as well as roles in proprioception and mechanotransduction along peripheral sensory neurons [58, 68, 69]. Accordingly, we hypothesize a proprioceptive role for *ppk23* during flight. *ppk23* ’s breadth of epistatic interactions suggests it plays a more central role in flight performance than previously expected. This includes epistatic interactions with genes identified in previous flight performance studies that were not found in our other analyses (*cac*, *Hk*, *Sh*, and *slo*) [70–73]. Pickpocket genes, including *ppk23*, may also play an important role in the central pattern generator (CPG) circuits of the thoracic ganglion, which acts as an important neuro-regulator of flight [74]. These circuits are responsible for ’fictive’ behavioral patterns (e.g. walking, flying, preening), or repetitive behaviors that can be maintained in the absence of sensory inputs. Fictive behaviors, such as the flight control phenotype we measured, can incorporate proprioceptive cues to modulate neuromuscular outputs that affect the organism’s behavior. Mutants of the pickpocket gene *ppk1* are known to affect larval locomotion by disrupting the timing of the output signals [75, 76]. As members of the degenerin/epithelial sodium channel (DEG/ENaC) family, pickpocket family genes may be important for promoting presynaptic homeostatic plasticity. Here, pickpocket genes create sodium ion "leaks" that depolarize the presynaptic membrane and promote calcium influx to aid in stabilizing neuronal function following the postsynaptic release of neurotransmitters [77, 78]. These pickpocket genes dimerize into larger pickpocket subunit assemblies, creating channels with different and unique physiological properties [77]. Thus, pickpocket genes, including *ppk23*, may also be important for ensuring motor neurons in flight musculature are functional throughout flight.

There was another noteworthy epistatic interaction between *ppk23* and *fruitless* (*fru*), an important transcription factor involved in differentiating sex-specific neural circuits that affects fitness-related behaviors, like courtship [79]. *fru* is also a modulator of CPG activity during flight [80], and is known to co-localize with *ppk23* in the thoracic ganglion [4, 58, 67, 81, 82]. Both also work with *doublesex* (*dsx*), another transcription factor that affects sex-specific neural circuits and was found in the whole gene, sex-difference analysis [83–85]. These genes suggest a potential genetic mechanism underlying the observed sexual dimorphism in flight performance (see S1 Text for more information on "Potential genetic sources of sexual dimorphism in flight performance").

### Natural variants in flight muscle genes associate with variation in flight performance

Two muscle-associated genes with known roles in flight were identified and validated from the additive screen. *Lasp* (human ortholog *LASP1*) modifies sarcomere and thin filament length, and myofibril diameter [52]; *bruno 1* (*bru1* or *aret*; human homolog *CLEF1* and *CLEF2*) is a transcription factor that controls alternative splicing of myofibrils in the indirect flight muscle [11, 53], among other developmental processes.

Interestingly, we also identified two genes affecting muscle function through PEGASUS_flies that were previously validated in the literature. *Tropomodulin* (*tmod;* human homolog *TMOD1*) is responsible for muscle function and *Glycerol-3-phosphate dehydrogenase 1* (*Gpdh1*; human homolog *GPD1*) affects metabolism within muscles [10, 86]. Neither gene contained a significant variant exceeding the additive screen’s significance threshold (*P* ≤ 1e-5), demonstrating PEGASUS_flies’ ability to identify genetic modifiers overlooked in a traditional minSNP approach.

### Variation in wing development contributes to variation in flight performance

One of the additive Bonferroni-corrected variants mapped to *Egfr*, a canonical developmental gene known to harbor natural variants that can modify wing morphology and flight performance [30, 31]. *Egfr* is pleiotropic, though one of the main roles it plays is in conjunction with the Bone Morphogenetic Protein (BMP) signaling pathway [13, 31, 87], an established pathway known to affect wing development. BMP signaling forms dose-dependent gradients influencing wing size, shape, and venation patterning [88–91]. It can also affect sensory and neuromuscular circuits in flight structures [8, 92]. In addition to our identification of several modifiers of BMP signaling across all analyses (*cmpy*, *Cul2*, *cv-2*, *cv-c*, *dally*, *daw*, *dpp*, *egr*, *gbb*, *hiw*, *kek5*, *Lis-1*, *Lpt*, *lqf*, *ltl*, *Mad*, *nmo*, *scw*, *Snoo*, *sog*, *srw*, *tkv*, *trio*), we functionally validated *Sno Oncogene* (*Snoo*; human homolog *SKI*), an important component of the BMP signaling pathway that affects dendritic tiling and morphogenesis, wing shape, and the development of sensory organs on the wing (e.g. microchaete and campaniform sensilla) [8, 50, 93, 94]. We also identified a significant marginal variant coding for a novel transcriptional start site in *short gastrulation* (*sog*; human homolog *Chordin*), a *dpp* antagonist in patterning the dorsoventral axis of the wings [89, 95, 96]. Additionally, *sog* is a known source of natural variants that modify flight performance in natural populations [13]. This particular *sog* variant had epistatic interactions in other genes containing marginal variants, including *ppk23* and *CG42671*, suggesting a more interconnected role for this antagonist of BMP signaling in modifying flight performance.

### Variation in gene regulation drives variation in flight performance

Similar to other DGRP studies [19, 29, 97], the majority of significant variants we had the power to test mapped to non-coding or intergenic regions, which are hypothesized to associate with cis-regulatory elements [29]. Under the omnigenic model, variation in these elements may have a disproportionate effect downstream when they occur in trans-regulatory genes (i.e. transcription factors, splicosomal proteins, and chromatin modifiers), since they themselves modify the regulation of other genes [32, 33]. One additive variant that passed the Bonferroni threshold mapped to an intronic region in *forkhead domain 96Ca* (*fd96Ca*; human homologs *FOXB1* and *FOXB2*), a *dorsal* (*dl*) transcription factor binding site (TFBS), and a silencer for *histone deacetylase 1* (*HDAC1*). *fd96Ca* is a forkhead box transcription factor expressed in neuroblasts along the longitudinal axis of the embryo and in some sensory neurons in the embryonic head [98]. Mis-regulation of transregulatory genes in developmental tissues (e.g. neuroblasts and the brain) may subsequently affect the ontogeny of tissues derived from it downstream. Another example highlighting the importance of trans-regulatory genes lies in the transcription factor CREG. It was initially selected as a negative control in the validation screen, though we demonstrate it has a significant effect on flight ability in our validation screen of candidate genes. The significant effect of the insert we tested in the screen may be a result of a difference between the insertional mutation and variation in the SNPs we have the power to test.

Further evidence supporting the role of regulatory elements affecting flight performance lies more broadly in our validation screen. The *Mi{ET1}* constructs introduced genetic variation into non-coding regions of genes, but not necessarily near the significant variants identified in our GWA screen. Thus, genetic variation across a gene, rather than at a single variant, is capable of affecting phenotypic variation. Our successful validation for 13/21 of the unique genes tested is near the approximately 70% success rate of other DGRP studies’ validation screens [29]. Interestingly, the insertion of the constructs into intronic regions both positively and negatively affected performance, even when done at independent sites in the same gene (*CadN* and *Dscam4*; see Fig 2C). Additionally, some candidate genes only validated in one sex, despite being identified in the sex-average analysis. These findings suggesting a more nuanced impact of genetic variation in cis-regulatory regions that can have differential sex-effects, possibly resulting from sexually-dimorphic epistatic interactions. Overall, their validation supports an important role for non-coding regions and regulation of gene expression, as well as the genes the constructs were inserted in, as modifiers of flight performance.

### A proposed model for understanding the genetic architecture of flight performance

Flight performance is likely an epiphenomenon of several interconnected complex traits. While we are unable to identify and validate every modifier, we likely identified the main features of the genetic architecture. Our results are biased, in part, by the annotations available for many of these genes. Many genes involved in the neural system are studied in the context of development, rather than their role in the adult stage. As a result, there is often relatively sparse information on their function in the adult fly. Our data suggests a means for studying these variants further in adults. Accordingly, we propose the following model to synthesize our findings primarily based on strongly significant genes we identified and validated, and supported by other genes we identified in the network and epistasis analyses (Fig 4C).

*Epidermal growth factor receptor* is a key gene in a canonical developmental pathway. It is a pleiotropic gene affecting wing morphology, sensory organ development, and neurodevelopment, on its own and through the BMP signaling pathway [31–33]. Proper development of these structures and circuits enables a well-connected peripheral nervous system to receive external, proprioceptive stimuli (through *CadN*, *Dscam4*, *fry*, *ppk23*, *Snoo*) and may also help shape the development of central pattern generator (CPG) circuits in the thoracic ganglion. As mentioned above, CPG circuits produce repetitive behavioral movements that may be modulated in either a sex-specific manner through *ppk23*, *fru*, and *dsx*, or a non-sex-specific manner with many other genes, including *ppk23* and other pickpocket family genes. The brain and thoracic ganglion process proprioceptive signals and activate motor neurons innervating the direct (control) and indirect (power) flight musculature (through *bru1*, *Gpdh1*, *Lasp*, *tmod*) at neuromuscular junctions. Activation of these muscles allows the properly developed wings to both flap and flip and generate lift [99]. The model proposed may help generate testable hypotheses that could help elucidate the architecture of flight as a complex trait.

## Conclusions

We highlight the benefits of conducting multiple computational analyses in evaluating variants, genes, and interaction networks and their contribution to the genetic architecture of flight performance by conducting a GWAS with the *Drosophila* Genetic Reference Panel lines. We also introduce PEGASUS_flies, an open-source and novel approach to whole gene significance screening. We present evidence that genetic modifiers with annotations for neurodevelopment, muscle and wing development, and regulation of other genes affect flight performance. We also uncover an important role for *ppk23* as an important epistatic hub in modulating flight performance, raising the importance of mechanosensation and proprioception in helping flies react and respond to an abrupt drop.

## Materials and methods

### Drosophila stocks and husbandry

All stocks were obtained from Bloomington *Drosophila* Stock Center (https://bdsc.indiana.edu/), including 197 *Drosophila* Genetic Reference Panel (DGRP) lines [17], 23 *Drosophila* Gene Disruption Project lines using the Mi{ET1} construct [41, 100], and two genetic background lines (w^1118^ and y^1^w^67c23^; S1 Table).

Flies were reared at 25° under a 12-h light-dark cycle. Stocks were density controlled and grown on a standard cornmeal media [101]. Two to three days post-eclosion, flies were sorted by sex under light CO_2_ anesthesia and given five days to recover before phenotyping.

### Flight performance assay

Flight performance was measured following the protocol refined by Babcock and Ganetzky [26]. Briefly, each sex-genotype combination consisted of approximately 100 flies, divided into groups of 20 flies across five glass *Drosophila* culture vials. These vials were gently tapped to draw flies down, and unplugged before a rapid inversion down a 25 cm chute. Vials stopped at the bottom, ejecting the flies into a 100 cm long x 13.5 cm diameter cylinder lined with a removable acrylic sheet coated in TangleTrap adhesive. Free falling flies instinctively right themselves before finding a place to land, which ended up immobilizing them at their respective landing height. Flies that passed through the column were caught in a pan of mineral oil and were excluded from the analysis.

After all vials in a run were released, the acrylic sheet was removed and pinned to a white poster board. A digital image was recorded on a fixed Raspberry PiCamera (V2) and the x,y coordinates of all flies were located with the ImageJ/FIJI Find Maxima function, set with a light background and noise tolerance of 30 [102]. For each sex-genotype combination, the mean landing height was calculated for only the flies that landed on the acrylic sheet.

### High-speed video capture of flight column

High-speed videos of flies leaving the flight column were recorded at 1540 frames per second using a Phantom Miro m340 camera recording at a resolution of 1920 x 1080 with an exposure of 150 μs (File 1 in https://doi.org/10.7910/DVN/ZTDHDC). The camera was equipped with a Nikon Micro NIKKOR (105 mm, 1:2.8D) lens and Veritas Constellation 120 light source.

### Estimating heritability

Individual fly landing heights were adjusted for the presence of inversions and Wolbachia status by sex and genotype, as calculated by the DGRP2 webserver. Using these adjusted landing heights by sex, we performed a random effects analysis of variance using the R (v.3.5.2) package lme4 (v.1.1.23): *Y ~ μ + L + ε*. Here, *Y* is the adjusted flight score, *μ* is the combined mean, *L* is the line mean, and *ε* is the residual. From this, sex-specific broad sense heritability (*H*^*2*^) estimates were calculated from the among line (*σ*_*L*_^2^) and error (*σ*_*E*_^2^) variance components: *H*^*2*^ = *σ*_*L*_^2^ / (*σ*_*L*_^2^ + *σ*_*E*_^2^).

### Genome wide association mapping

Flight performance scores for males and females were submitted to the DGRP2 GWAS pipeline (http://dgrp2.gnets.ncsu.edu/) [16, 17] and results for each sex, and the average (sex-average) and difference (sex-difference) between them were all considered (S2 Table). In total, 1,901,174 variants with a minor allele frequency (MAF) ≥ 0.05 were analyzed (File 2 in https://doi.org/10.7910/DVN/ZTDHDC). All reported additive variant *P*-values result from a linear mixed model analysis, including *Wolbachia* infection and presence of five major inversions as covariates. Variants were filtered for significance using the conventional *P* ≤ 1e-5 threshold [29]. Effect size estimates were calculated as one-half the difference between the mean landing heights for lines homozygous for the major vs. minor allele. The contribution of individual variants to the overall effects was estimated as the absolute value of an individual variant’s effect size divided by the sum of the absolute values for all conventionally significant (*P* ≤ 1e-5) variants’ effect sizes.

### Candidate gene disruption screen

Candidate genes were validated using insertional mutant stocks generated from Gene Disruption Project [42]. These stocks contain a *Minos* enhancer trap construct Mi{ET1} [41] and were built on either w^1118^ or y^1^ w^67c23^ backgrounds (BDSC_6326 and BDSC_6599, respectively).

Control and experiment line genetic backgrounds were isogenized with five successive rounds of backcrossing the insertional mutant line to its respective control. Validation of flight phenotypes was done using offspring of single-pair (1M x 1F) crosses between the control and insert lines. Heterozygous flies from these crosses were mated in pairs and the homozygous offspring lacking the insertion were collected as the control. Candidate heterozygous/homozygous positive lines were mated as pairs once more and lines producing only homozygous positive offspring were used as experimental lines (S1 Fig). Experimental lines were checked for a GFP reporter three generations later to confirm their genotype. The finalized recombinant backcrossed control and experimental lines for each sex-genotype combination were assayed for flight performance, and tested for significance, via Mann-Whitney U-tests.

### Calculating gene-score significance

Gene-scores were calculated using Precise, Efficient Gene Association Score Using SNPs (PEGASUS) [25]. Originally implemented with human datasets, we modified the program to work with *Drosophila* datasets, which we call PEGASUS_flies. It also contains default values adjusted for *Drosophila*, a linkage disequilibrium file, and gene annotations drawn from the FB5.57 annotation file, available on the DGRP webserver. PEGASUS_flies is available at: https://github.com/ramachandran-lab/PEGASUS_flies, and as File 4 in https://doi.org/10.7910/DVN/ZTDHDC.

### Identifying altered sub-networks of gene-gene and protein-protein interaction networks

Returned gene-scores were filtered for genes of high confidence using the Twilight package (v.1.60.0) in R (Scheid and Spang 2005). Here, we estimated the local False Discovery Rate (lFDR) of all previously output gene scores using the *twilight* function. Taking the inflection point of the (1—lFDR) curve, our high-confidence gene scores ranged from 0.65–0.73 for the four, sex-based phenotypes (S8 Table). High confidence genes were–log10 transformed, while the remaining were set to 0.

Hierarchical HotNet was used to identify altered sub-networks of interacting genes or proteins [55] based on network topology generated from several gene-gene or protein-protein interaction networks. The four adjusted, sex-based gene-score vectors were mapped in the program to fifteen interaction networks obtained from High-quality INTeractomes (HINT) [103], the *Drosophila* Interactions Database (Droidb) [104, 105], and the *Drosophila* RNA^i^ Screening Center (DRSC) Integrative Ortholog Prediction Tool (DIOPT) [106]. Consensus networks were calculated from 100 permutations of all four gene-score vectors on each of the fifteen interaction networks and filtered to include at least three members. The largest sub-network and the remaining eight sub-networks were passed to Gene Ontology enRIchment anaLysis and visuaLizAtion tool (GOrilla) to identify enrichment for gene ontology (GO) categories [107, 108].

### Screening for epistatic interactions

Epistatic hub genes were identified using MArginal ePIstasis Test (MAPIT), a linear mixed modeling approach that tests the significance of each SNP’s marginal effect on a chosen phenotype. MAPIT requires a complete genotype matrix, without missing data. SNPs were imputed using BEAGLE 4.1 [109, 110] and then filtered for MAF ≥ 0.05 using VCFtools (v.0.1.16) [111]. MAPIT was run using the Davies method on the imputed genome (File 2 in https://doi.org/10.7910/DVN/ZTDHDC), DGRP2 webserver-adjusted phenotype scores for each sex-based phenotype (S2 Table), DGRP2 relatedness matrix and covariate file containing *Wolbachia* infection and the presence of five major inversions [17].

Resulting marginal effect *P*-values (File 3 in https://doi.org/10.7910/DVN/ZTDHDC) were filtered to a Bonferroni threshold (*P* ≤ 2.56e-8) and tested for pairwise epistatic interactions in a set-by-all framework against the initial 1,901,174 SNPs (unimputed; MAF ≥ 0.05) using the PLINK–epistasis flag (v.1.90) [112]. Results were filtered for all *P*-values that exceeded a Bonferroni threshold, calculated as 0.05 / (the number of Bonferroni marginal effect *P*-values x 1,901,174 SNPs).

### Annotating FBgn and orthologs

Annotations and unreferenced descriptors of gene functions, expression profiles, and orthologs were gathered from autogenerated summaries on FlyBase [38, 39]. These summaries and descriptors were compiled from data supplied by the Gene Ontology Consortium [27, 28], the Berkeley *Drosophila* Genome Project [113], FlyAtlas [114], The Alliance of Genome Resources Consortium [115], modENCODE [38], PAINT [116], the DRSC Integrative Ortholog Prediction Tool (DIOPT) [106], and several transcriptomics and proteomic datasets [11, 12, 43, 114, 117–119].

Flybase gene (FBgn) identifiers were converted to their respective *D*. *melanogaster* (Dmel) or *H*. *sapiens* (Hsap) gene symbols using the *Drosophila* RNA^i^ Stock Center (DRSC) Integrative Ortholog Prediction Tool (DIOPT) [106]. FBgn were filtered for all high to moderate confidence genes, or low confidence genes if they contained the best forward and reverse score.

### Calculating an empirically simulated significance threshold

In order to identify any spurious associations between the DGRP genotyped lines and the four phenotypes of interest, we performed a permutation-based test using the—mperm flag in PLINK v.1.90. For each phenotype, we performed 10,000 permutations of the phenotype values across lines and tested those randomly assigned values for an association to the permuted phenotype. We find that all of the variants associated with the four phenotypes in the standard GWAS framework remained significant after filtering based on the permutation p-value (*P* < 0.05).

### GO term analysis

GOWINDA [40] was implemented to perform a Gene Ontology (GO) analysis that corrects for gene size in GWA studies. We conducted this analysis for male (n = 418), female (n = 473), sex-average (n = 527), and sex-difference (n = 214) candidate SNPs exceeding a relaxed *P* < 1e-4 significance threshold, against the 1,901,174 SNPs with MAF ≥ 0.05. We ran 100,000 simulations of GOWINDA using the gene mode and including all SNPs within 2000 bp.

Gene Ontology enRIchment anaLysis and visuaLizAtion tool (GOrilla) [107, 108] was run on PEGASUS_flies gene-scores and Hierarchical Hotnet sub-networks using the default commands and a gene list compiled from all genes available in the FB5.57 annotation file.

### Weighted Gene Co-expression Network Analysis

To test whether ambient adult transcriptomes could explain the observed phenotypic variation, we turned to the publicly available DGRP2 microarray data, downloaded from the DGRP2 webserver [17]. These data represent the transcriptomes for untreated young adult flies of each sex. We performed Weighted Gene Co-expression Network Analysis (WGCNA) analyses using the WGCNA R package [59] to cluster and correlate the expression profiles of genes from 177 shared, DGRP lines. This analysis was run using the following parameters: power = 16 (from soft threshold analysis ≥ 0.9), merging threshold = 0.0, signed network type, maximum blocksize = 1000, minimum module size = 30.

## Supporting information

S1 Fig DGRP lines’ mean flight performance is highly repeatable across generations. Set of genotypes (n = 12) reared 10 generations apart show very strong agreement (r = 0.95) in mean flight performance scores. The regression line (red line) through the point pairs (black points) has nearly the same slope and y-intercept as the y = x line (gray dashed line).
(TIF)

S2 Fig Sex-average and sex-difference phenotypic distributions are amenable to an association study. Distribution in mean landing height (m) for (A) sex-average and (B) sex-difference phenotypes suggest ample phenotypic variation exists to run an association study. Each plot is sorted in order of increasing phenotype score, independent of one another.
(TIF)

S3 Fig QQ-plots show enrichment for some additive variants across each of the sex-based phenotypes. Plots comparing the theoretical vs. observed *P*-value distribution across (A) males, (B) females, (C) sex-average, and (D) sex-difference phenotypes. Red line denotes y = x.
(TIF)

S4 Fig Top additive associations are spaced throughout the genome. Top additive variants, those reported in DGRP2 webserver file with the `top.annot`suffix, are largely free of linkage blocks. There is a larger block on X, corresponding with 10 variants that map to an intron and one synonymous coding site in *CG32506*. The heat component corresponds with likelihood of that variant being in a linkage block from less (0—blue) to more likely (1—red).
(TIF)

S5 Fig Additional sex-based phenotype Manhattan plots for additive analysis. (A) Males, (B) females, and (C) sex-difference phenotypes all have significant additive variants (red points) pass a traditional DGRP threshold (*P* ≤ 1e-5, gray solid line), and at least one variant passes a Bonferroni threshold (*P* ≤ 2.63e-8, gray dashed line, red dot with black outline). Variants are arranged in order of relative genomic position by chromosome and plotted by the–log10 of the *P*-value. The sex-average panel is displayed in text.
(TIF)

S6 Fig Genetic crosses performed for deriving experimental and control stocks used to validate candidate genes. All crosses are represented with females on the left and males on the right. Ten single pair crosses of a female genetic control, either w^1118^ (pictured) or y[1] w[67c23], in white boxes were crossed with the respective *Mi{ET1}* insertional mutant line in green boxes. After the initial cross, heterozygous flies were backcrossed to the respective genetic control for five generations. In the sixth generation, single pairs of heterozygous flies were crossed. Progeny without the Avic\GFP^E.3xP3^ marker were collected as homozygous nulls, while several vials of putatively homozygous mutants (no progeny without marker) were crossed again to confirm genotype. Stocks were monitored for two additional generations to confirm mutant carrier status before a homozygous mutant stock was selected as an experimental line.
(TIF)

S7 Fig Significant whole genes are distributed throughout the genome and sex-based phenotypes. Whole gene analyses conducted with PEGASUS_flies for (A) males, (B) females, and (C) sex-difference phenotypes showed enrichment for significant whole genes across these three, and the sex-average (displayed in text). Each dot represents a whole gene, ordered by position across the chromosomes and plotted as the–log10 of the gene-score. Points above the Bonferroni threshold (*P* ≤ 3.03e-6, gray line) are colored in red.
(TIF)

S8 Fig Significant marginal variants are unevenly distributed across sex-based phenotypes. (A) Males had very few significant variants (red points) pass a Bonferroni threshold (*P* ≤ 2.56e-8, gray solid line), while (B) females had more and (C) sex-average had the most. (D) Sex-difference had no significant marginal variants. Variants are arranged in order of relative genomic position by chromosome and significance scores–log10 transformed.
(TIF)

S9 Fig Trait-relationship correlation matrix shows no correlation between measured phenotypes and young adult transcriptome. Neither sexes’ mean landing height, standard deviation in landing height, or proportion of flies that fell through the column (fallen) were significant with a cluster of similarly expressed genes in a Weighted Gene Co-expression Network Analysis (WGCNA). Colored modules on the left represent WGCNA-generated clusters of genes and the color of each table cell corresponds with the magnitude of correlation coefficient (top number in cell). The bottom number in each cell is the significance of the correlation. No clusters were significantly correlated with any sex-phenotype combination.
(TIF)

S1 Table *Drosophila* stocks used in this study. (XLSX)

S2 Table Raw and adjusted flight performance metrics. (XLSX)

S3 Table No significant correlations were observed between flight performance and other DGRP phenotypes. (XLSX)

S4 Table Up to two inversions were significant covariates in three of the sex-based analyses. (XLSX)

S5 Table Several additive variants associated with flight performance. (XLSX)

S6 Table Several candidate genes were validated for flight performance. (XLSX)

S7 Table Several gene-scores pass a Bonferroni threshold across all four sex-based phenotypes. (XLSX)

S8 Table Twilight-estimated local False Discovery Rate (lFDR) cutoff thresholds for PEGASUS_flies gene-scores. (XLSX)

S9 Table Hierarchical HotNet sub-network gene annotations. (XLSX)

S10 Table Large sub-network from Hierarchical HotNet is enriched for trans-regulatory factors and neurodevelopmental Gene Ontology terms. (XLSX)

S11 Table Collection of smaller sub-networks from Hierarchical HotNet are collectively enriched for mRNA splicing and autophagy Gene Ontology terms. (XLSX)

S12 Table Significant marginal variants identified from MAginal ePIstasis Test (MAPIT). (XLSX)

S13 Table Epistatic interactions play a large role in shaping the genetic architecture of flight performance. (XLSX)

S14 Table Epistatic interactions with *pickpocket 23* (*ppk23*) are enriched in a Gene Ontology (GO) term analysis form neurodevelopmental genes. (XLSX)

S15 Table Genes mapped to from epistatic interactions with *CG42671* are significantly enriched for neurodevelopment in a Gene Ontology (GO) analysis. (XLSX)

S16 Table Gene set enrichment analysis for significant epistatic interactors within a 669 bp intergenic region between chr3L:6890373–6891042 suggests enrichment for neurodevelopmental Gene Ontology categories. (XLSX)

S17 Table Master lookup table for all genes identified. (XLSX)

S1 Text Supplemental results. (DOCX)
