# Supplementary figures and images for: Natural variation in the regulation of neurodevelopmental genes modifies flight performance in Drosophila
Source: PLoS Genet. 2021 Mar 18;17(3):e1008887. doi: 10.1371/journal.pgen.1008887 (PMC7971549; doi:10.1371/journal.pgen.1008887)

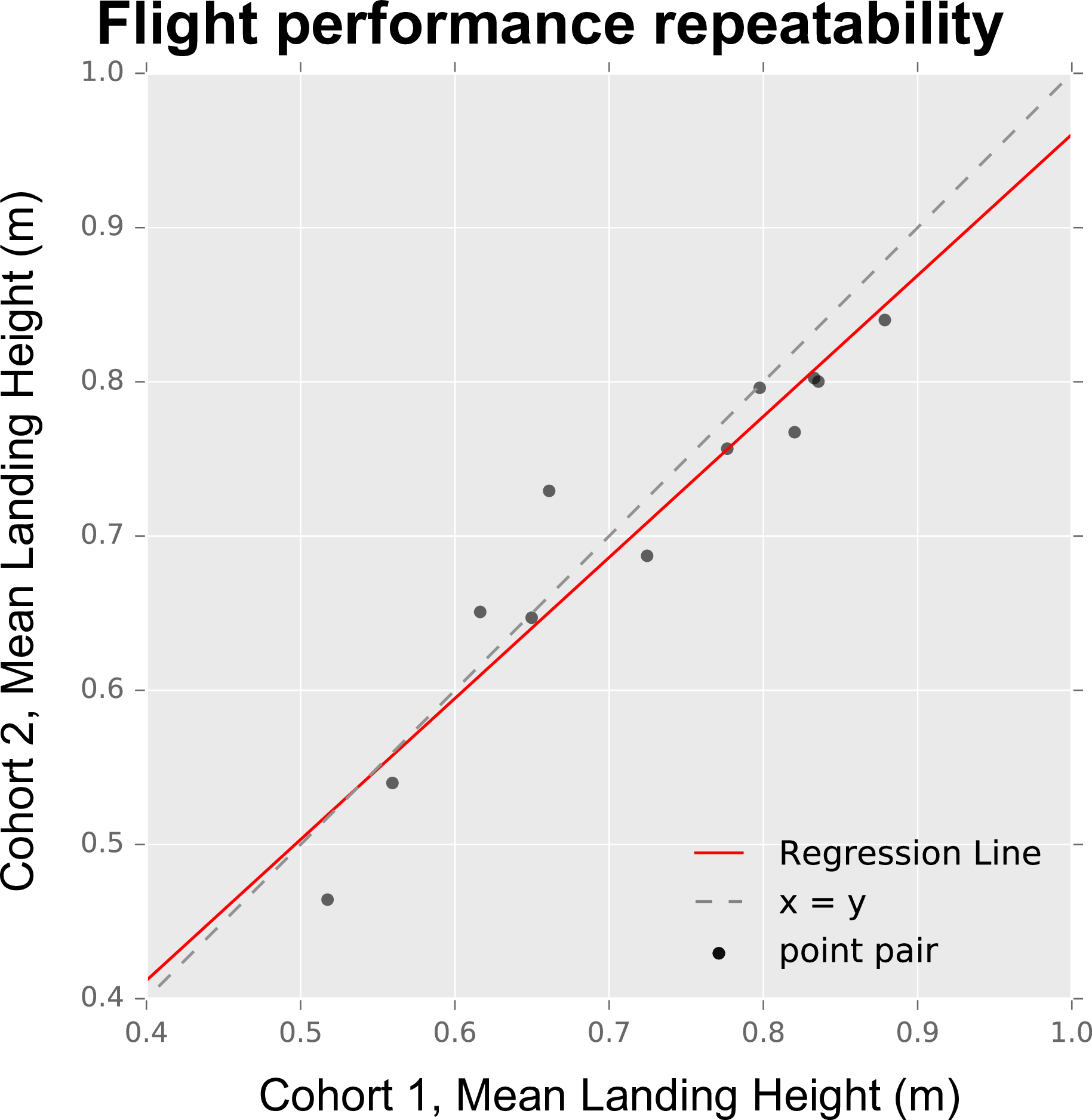

Supplement: S1 Fig — Set of genotypes (n = 12) reared 10 generations apart show very strong agreement (r = 0.95) in mean flight performance scores. The regression line (red line) through the point pairs (black points) has nearly the same slope and y-intercept as the y = x line (gray dashed line). (TIF) [file pgen.1008887.s001.tif]

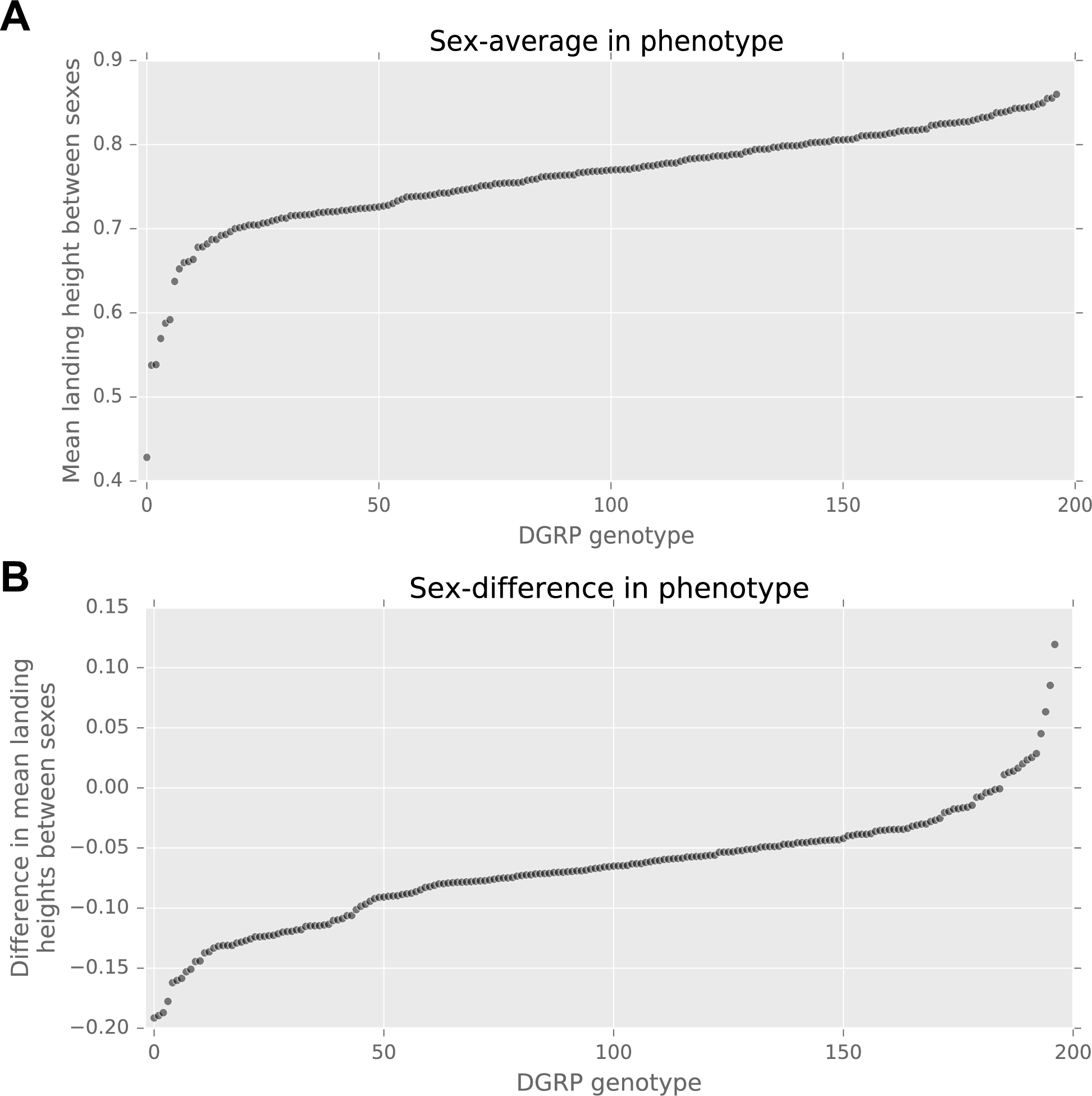

Supplement: S2 Fig — Distribution in mean landing height (m) for (A) sex-average and (B) sex-difference phenotypes suggest ample phenotypic variation exists to run an association study. Each plot is sorted in order of increasing phenotype score, independent of one another. (TIF) [file pgen.1008887.s002.tif]

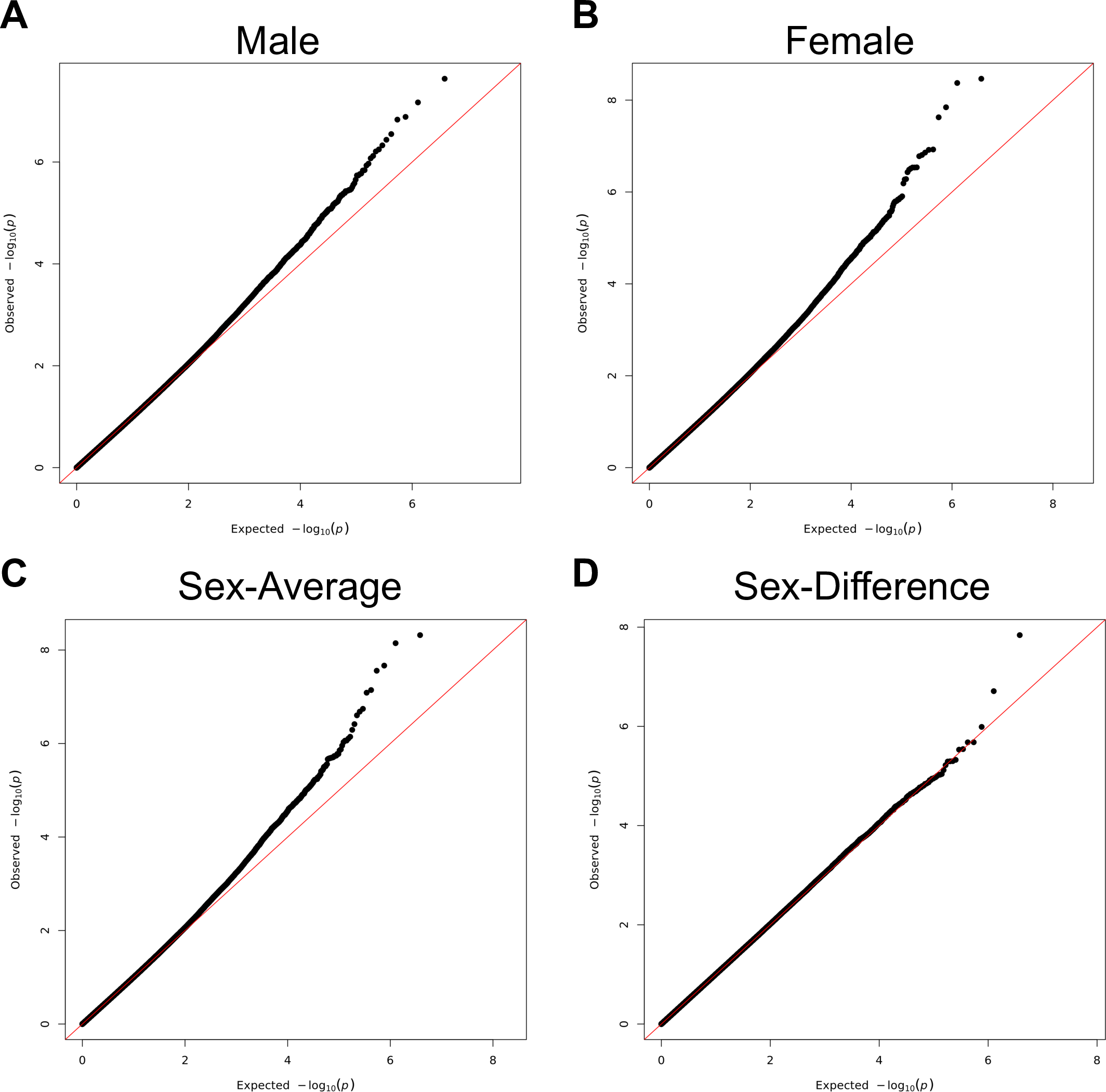

Supplement: S3 Fig — Plots comparing the theoretical vs. observed P-value distribution across (A) males, (B) females, (C) sex-average, and (D) sex-difference phenotypes. Red line denotes y = x. (TIF) [file pgen.1008887.s003.tif]

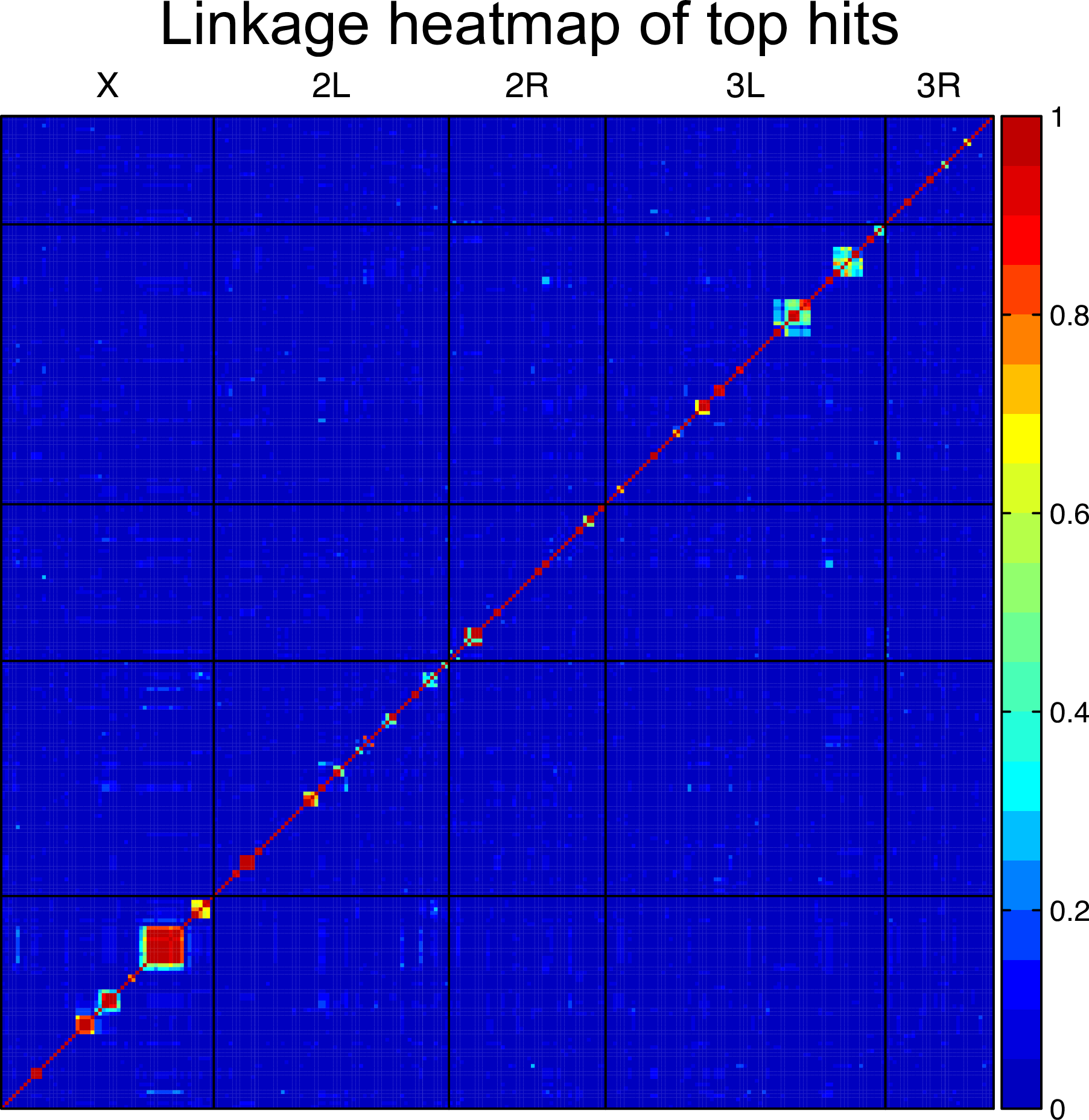

Supplement: S4 Fig — Top additive variants, those reported in DGRP2 webserver file with the `top.annot`suffix, are largely free of linkage blocks. There is a larger block on X, corresponding with 10 variants that map to an intron and one synonymous coding site in CG32506. The heat component corresponds with likelihood of that variant being in a linkage block from less (0—blue) to more likely (1—red). (TIF) [file pgen.1008887.s004.tif]

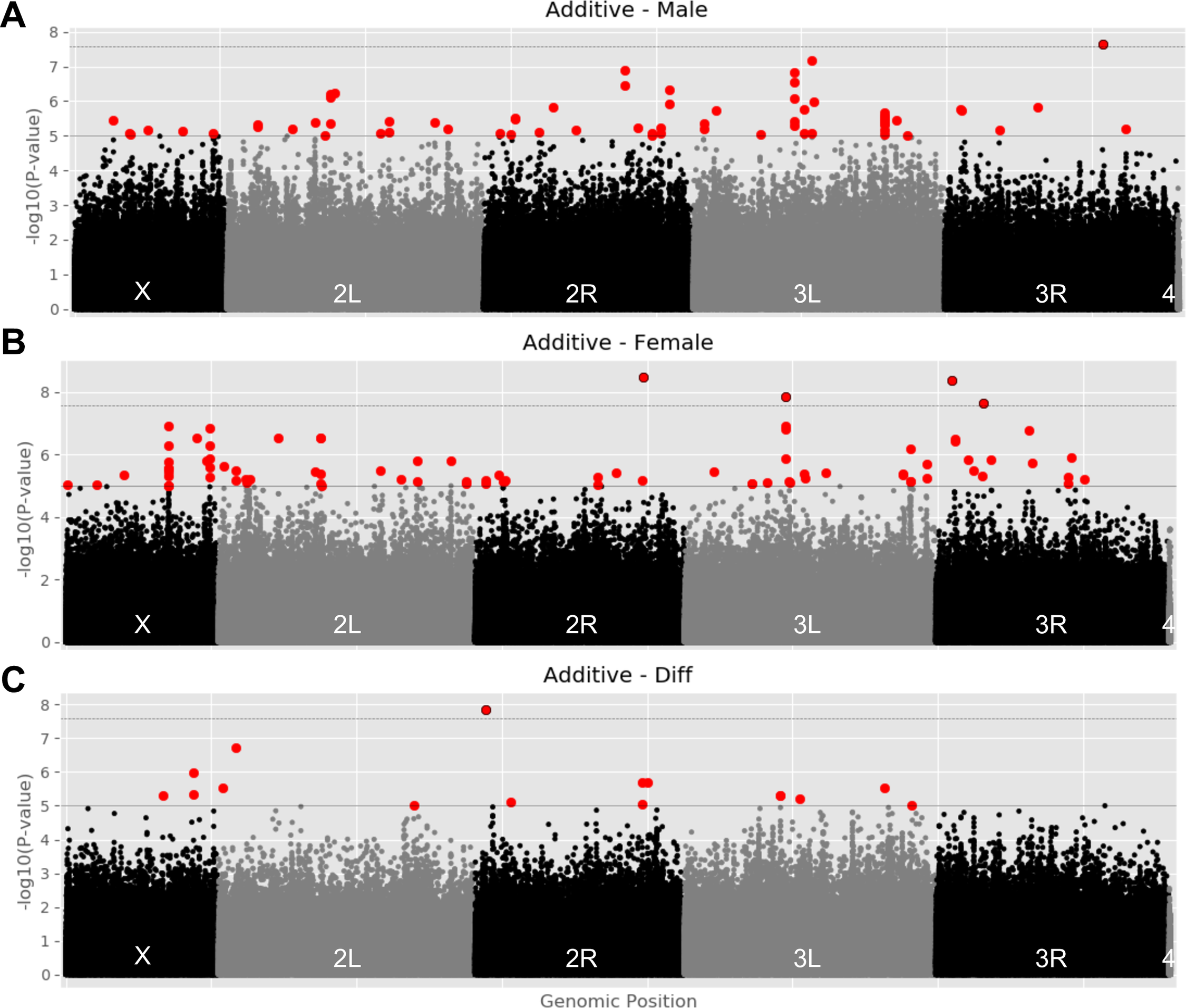

Supplement: S5 Fig — (A) Males, (B) females, and (C) sex-difference phenotypes all have significant additive variants (red points) pass a traditional DGRP threshold (P ≤ 1e-5, gray solid line), and at least one variant passes a Bonferroni threshold (P ≤ 2.63e-8, gray dashed line, red dot with black outline). Variants are arranged in order of relative genomic position by chromosome and plotted by the–log10 of the P-value. The sex-average panel is displayed in text. (TIF) [file pgen.1008887.s005.tif]

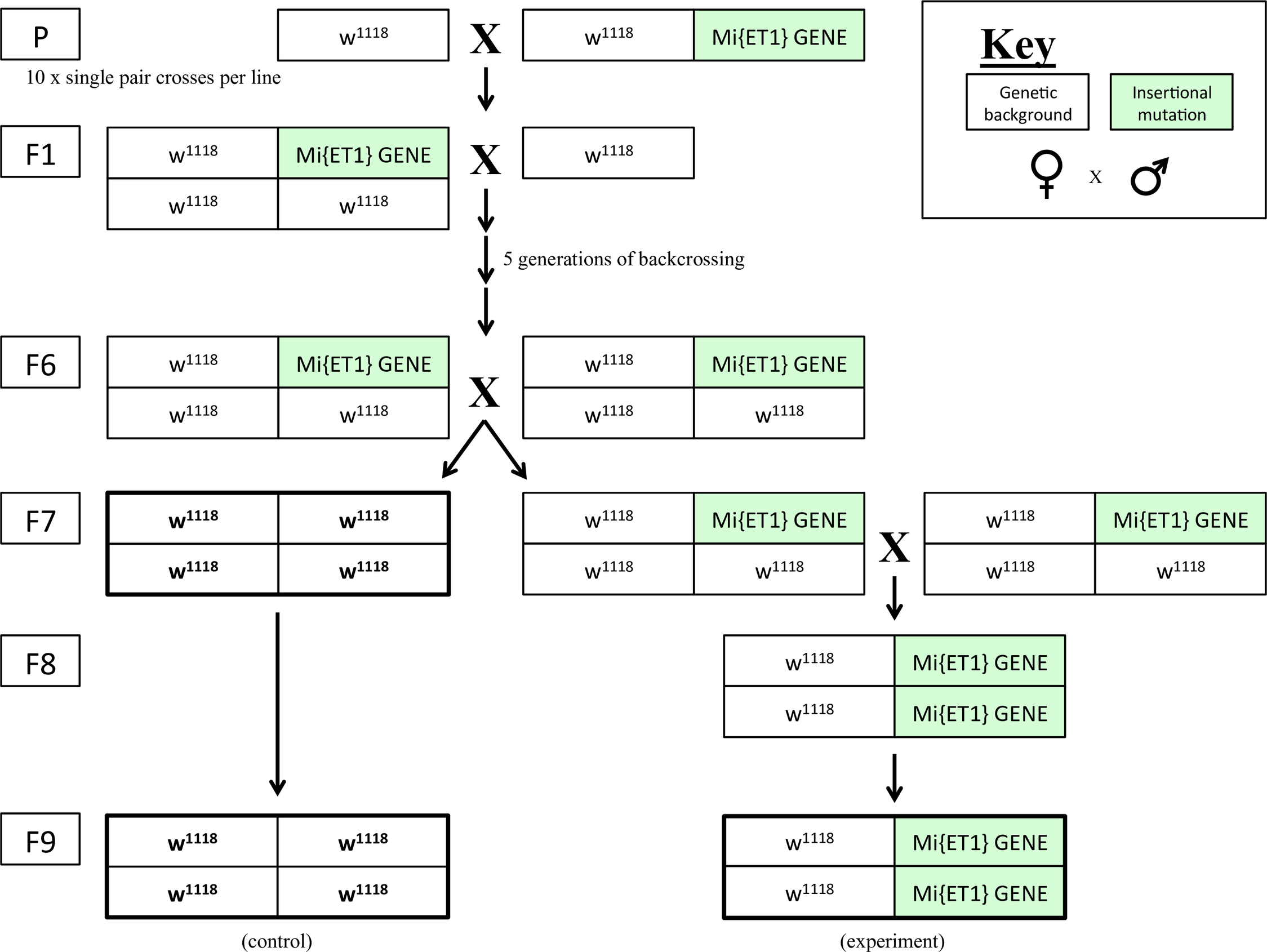

Supplement: S6 Fig — All crosses are represented with females on the left and males on the right. Ten single pair crosses of a female genetic control, either w1118 (pictured) or y[1] w[67c23], in white boxes were crossed with the respective Mi{ET1} insertional mutant line in green boxes. After the initial cross, heterozygous flies were backcrossed to the respective genetic control for five generations. In the sixth generation, single pairs of heterozygous flies were crossed. Progeny without the Avic\GFPE.3xP3 marker were collected as homozygous nulls, while several vials of putatively homozygous mutants (no progeny without marker) were crossed again to confirm genotype. Stocks were monitored for two additional generations to confirm mutant carrier status before a homozygous mutant stock was selected as an experimental line. (TIF) [file pgen.1008887.s006.tif]

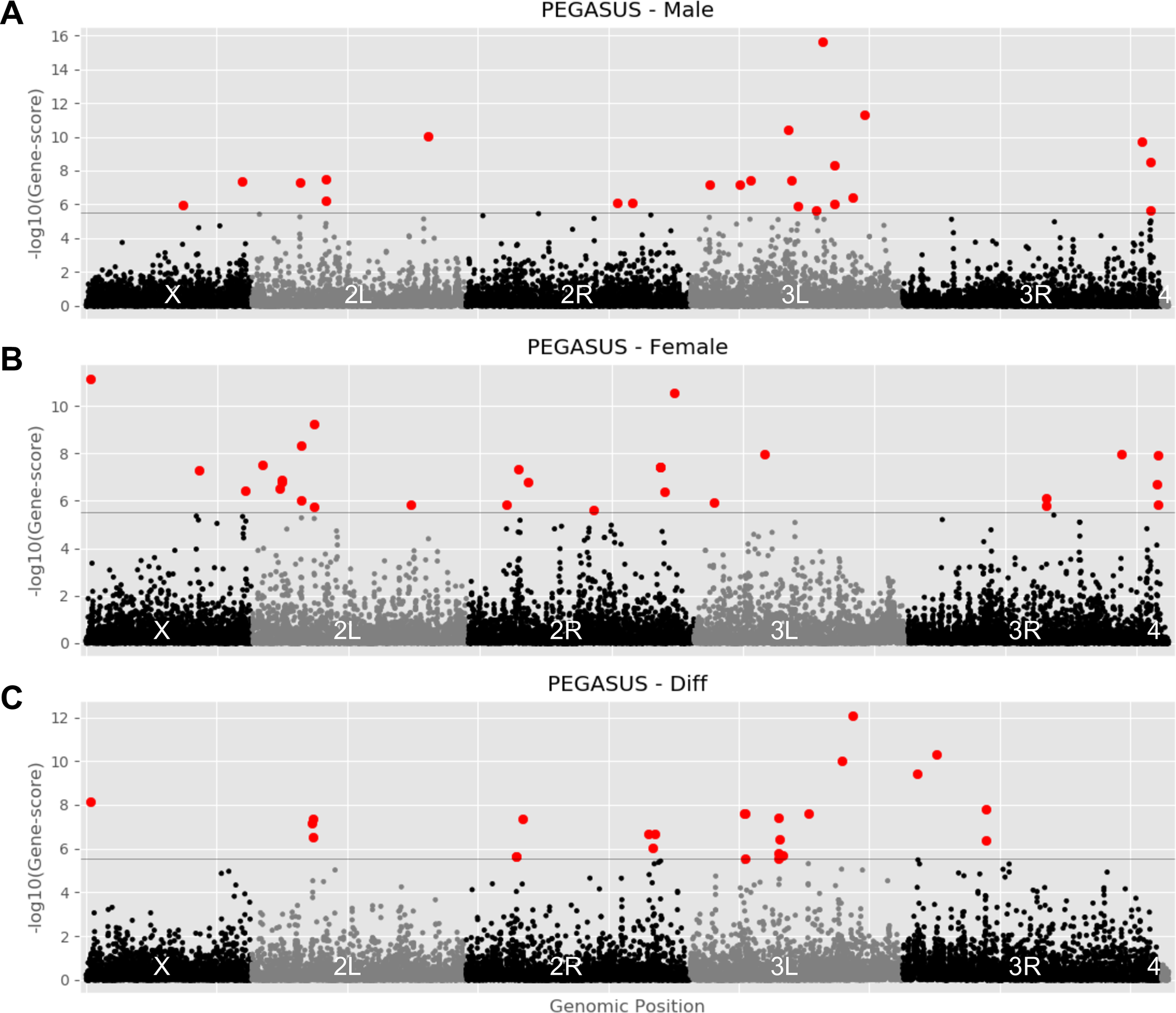

Supplement: S7 Fig — Whole gene analyses conducted with PEGASUS_flies for (A) males, (B) females, and (C) sex-difference phenotypes showed enrichment for significant whole genes across these three, and the sex-average (displayed in text). Each dot represents a whole gene, ordered by position across the chromosomes and plotted as the–log10 of the gene-score. Points above the Bonferroni threshold (P ≤ 3.03e-6, gray line) are colored in red. (TIF) [file pgen.1008887.s007.tif]

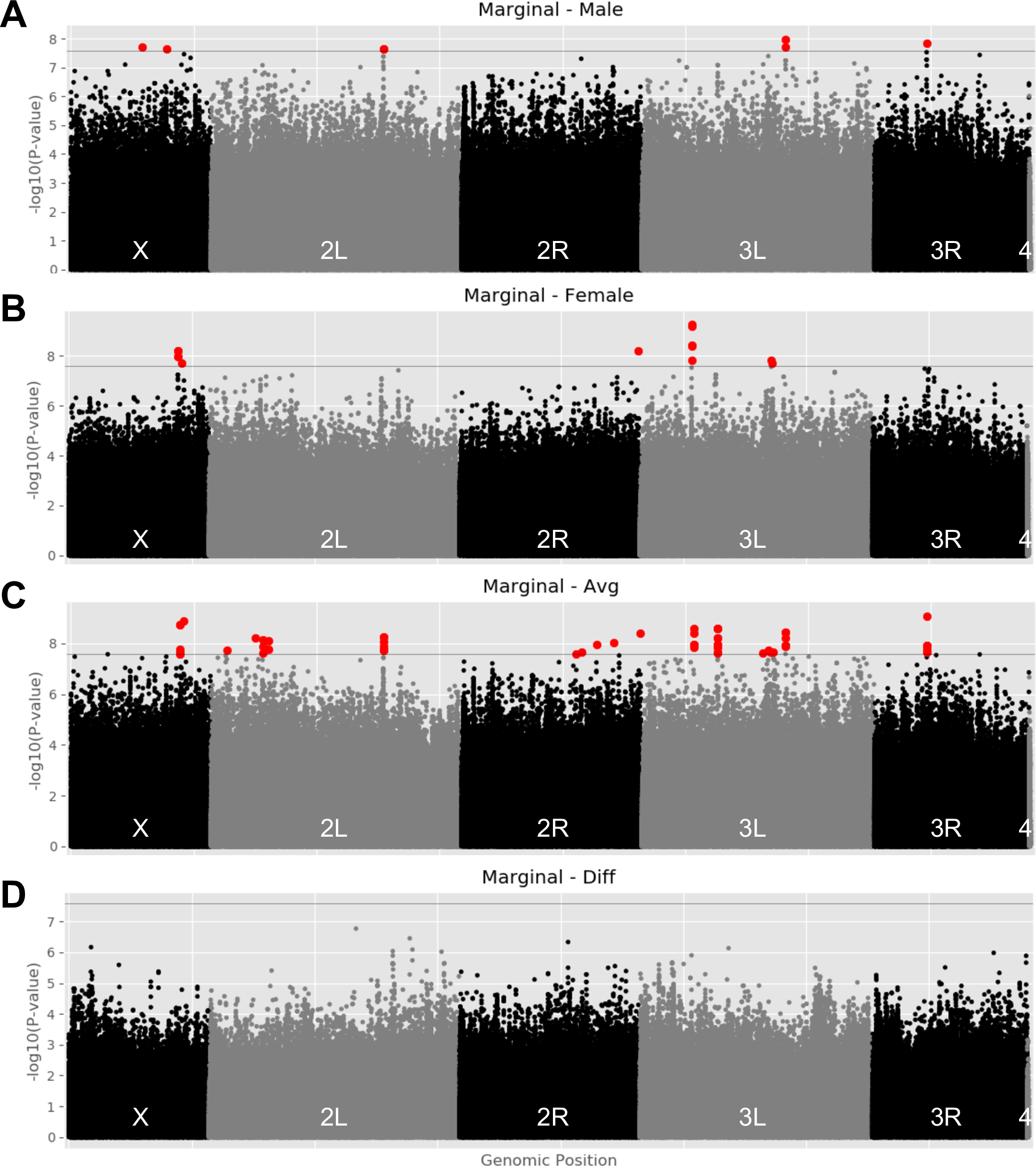

Supplement: S8 Fig — (A) Males had very few significant variants (red points) pass a Bonferroni threshold (P ≤ 2.56e-8, gray solid line), while (B) females had more and (C) sex-average had the most. (D) Sex-difference had no significant marginal variants. Variants are arranged in order of relative genomic position by chromosome and significance scores–log10 transformed. (TIF) [file pgen.1008887.s008.tif]

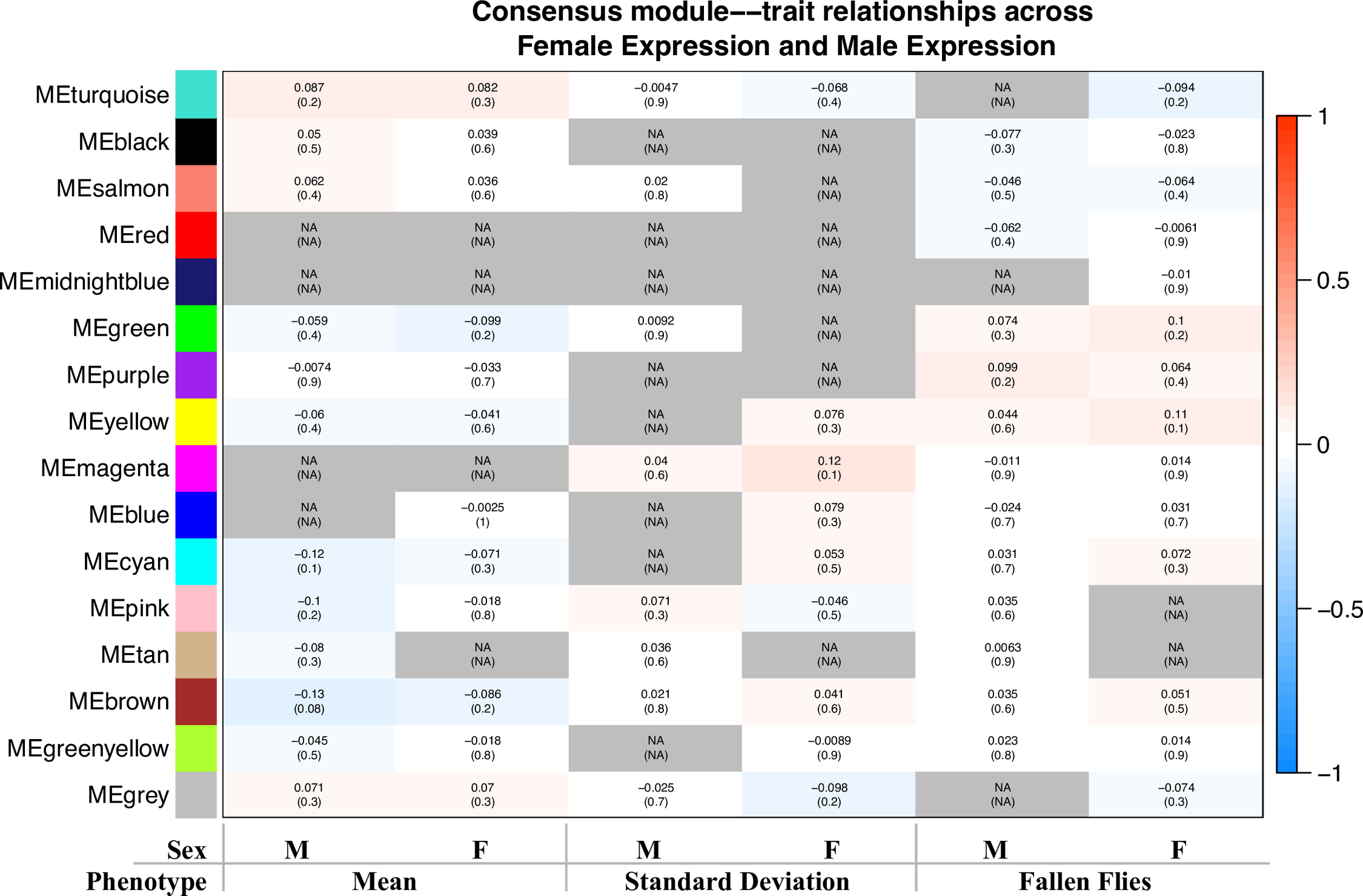

Supplement: S9 Fig — Neither sexes’ mean landing height, standard deviation in landing height, or proportion of flies that fell through the column (fallen) were significant with a cluster of similarly expressed genes in a Weighted Gene Co-expression Network Analysis (WGCNA). Colored modules on the left represent WGCNA-generated clusters of genes and the color of each table cell corresponds with the magnitude of correlation coefficient (top number in cell). The bottom number in each cell is the significance of the correlation. No clusters were significantly correlated with any sex-phenotype combination. (TIF) [file pgen.1008887.s009.tif]
