# Supplementary material for: Natural variation in the regulation of neurodevelopmental genes modifies flight performance in Drosophila
Source: PLoS Genet. 2021 Mar 18;17(3):e1008887. doi: 10.1371/journal.pgen.1008887 (PMC7971549; doi:10.1371/journal.pgen.1008887)
Supplement: S1 Text — (DOCX) [file pgen.1008887.s027.docx]

**Supplemental Results**

**Putative roles for *flippy* and *flapper***

We functionally validated two unnamed candidate genes and are renaming them *flippy* (*flip*, formerly *CG9766*) and *flapper* (*flap*, formerly *CG11073*). Only tangential evidence of their functions exists, though both have evidence of pleiotropy.

*flippy* (human homolog *FANK1*) has important roles in neuroanatomical development [1,2] and sperm development [3]. It is important in the development of trichogen cells, which are precursors to the chaete flies use for mechanosensation. In humans, *FANK1* plays roles in spermatogenesis and apoptosis, and is a putative evolutionary target of balancing selection [4,5]. Given *flippy*’s pleiotropic role in neurodevelopment and gametogenesis, it may also be under stabilizing selection brought about by contrasting selective pressures for neural function and fitness.

*flapper* is expressed in the peripodial epithelium cells of the eye, leg, and wing imaginal discs [6]. It is expressed at very high levels during 16-18 hours of embryogenesis, pupariation [7] and in the head, eyes, and carcass in the adult stage [8]. It was previously identified as a candidate gene in a screen for modifiers of circadian rhythm [9] and was significantly upregulated in flies bred for aggressive behavior [10], however both studies failed to functionally validate the gene. *flapper* was also implicated in the downregulation of amyloid-β peptides [11] and in late life fecundity [12] suggesting it may play a basic role in development that affects several phenotypes. Accordingly, we hypothesize it plays some role in patterning neural circuitry of sensory neurons on the cuticle and eyes, and facilitates neural circuit assembly in the brain.

**Association of epistatic interactions with flight performance, continued**

In females, one intronic SNP (3L_11217593_SNP) mapped to *CG42671.* Little is known about this gene and there are no human orthologs, but we can gain insights into its function based on the 51 epistatic variants that mapped to 37 genes with annotations for regulation of gene expression (*arx*, *bi*, *CG6843*, *Ches-1-like*, *dve*, *HDAC1*, *Moe*, *RpL26, Sdc*, *Tgi*), and neural development, signaling, and function (*cact, CG13579, HDAC1, ed, ngl3, nrm, numb, Sdc*). Four other variants (X_17459818_SNP, X_17459830_SNP, X_17460743_DEL, X_17460820_SNP) mapped to a 1,002 bp region downstream of *pickpocket 23* (*ppk23*). These marginal variants significantly interacted with 2,162 other variants, which mapped to 1,042 genes. Many of these genes were also found in the sex-average analysis.

The sex-average phenotype had the greatest number of significant marginal variants (62; 11 also found in females) and epistatic interactions. The following paragraphs describe these findings. Seven variants mapped to *ppk23*, the most of any individual gene. These variants interacted with 4,895 variants across 2,010 unique genes, which collectively had significant GO term enrichment for neuronal growth, organization and differentiation (S14 Table). One of these genes was *CG42671,* which was also present as a significant marginal variant in the female and sex-average epistasis screens. In the sex-average epistasis screen, *CG42671* interacted with 1,013 variants across 616 genes, which were significantly enriched in a gene set enrichment analysis for neurodevelopment, particularly neuron growth and movement (S15 Table). *CG42671* is understudied and lacks substantive annotations, but based on its interactors’ significant GO categories, *CG42671* is likely involved in growth and neuronal target finding.

Separately, *CG10936* contained a significant marginal variant. The gene is poorly annotated, though it was previously identified in a screen for nociception [13]. It had epistatic interactions with 29 genes annotated for neurogenesis and function (*CG42788*, *Dh31*, *fru, hiw,* *lilli*, *nAChRalpha4*), as well as regulation of gene expression through chromatin modification (*Etl1* and *lilli*) and alternative splicing (*Srp54* and *U2af38*).

One variant (2R_16871314_SNP) overlapped with two gene windows: the 3’ UTR of *CG9313* and 29 bp downstream of *CG15651*. *CG9313* (orthologous to human *DNAI1*) is an ATP-dependent microtubule motor and is involved in sperm development and the sensory perception of sound in *Drosophila* and proprioception [14]. *CG15651* is predicted to localize to the rough endoplasmic reticulum and Golgi body during embryogenesis, early larval, and late pupation stages where it is expressed in the central nervous system. Its human ortholog, *FKRP* (fukutin related protein), is implicated in intellectual disability and it is a candidate gene therapy target for muscular dystrophy [15-17]. These genes’ shared function in nervous system development is reflected in the variants that map to 87 genes with annotations for neuron development, patterning, and function (*5-HT2B, cwo, dally, dx, Dysb*, *enok*, *erm, mbl, Ngl1, nmo, Sdc, Sema1a, sNPF*, *tup*). Several genes were also annotated for endoplasmic reticulum function (*bark*, *CG5885*, *CG15651*, *Fatp3*, *PAPLA1*, *Trc8*, *Uggt*); chromatin remodeling (*CG43902, enok, erm, lncRNA:roX1*, *tim*); transcription and alternative splicing (*cwo, bru3, CG6841, CG9650, CG15710, enok, luna, mbl, tim, tup*); and gene product regulation (*bru3, cwo, CG5885, CG9650, CG15710, luna, tRNA:Arg-TCT-2-1, tup*).

Finally, there were six intergenic variants in a 669 bp region (chr3L:6890373 - 6891042). This region lacked regulatory annotations, yet collectively interacted with 513 variants mapping to 309 genes. Many of these interacting genes also interacted with *ppk23*, *CG42671*, and *CG10936*. Similarly, these genes had significant GO term enrichment for neurodevelopment and neuron function (S16 Table). While some of these genes are poorly studied, their putative interactions can shed light on potential functions and implications in broader genetic networks. They also speak to the highly interconnected nature of the significant genes in this study in influencing phenotype.

**Potential genetic sources of sexual dimorphism in flight performance**

The interconnectedness of *ppk23* also provides clues about the sexual dimorphism observed in flight. While males generally outperform females, likely due to differences in weight, sex failed to explain ~25% of the variation between the two groups. Like most pickpocket family genes, *ppk23* is well established as an important factor in highly sex-specific phenotypes, such as pheromone detection and courtship [18-20]. One of *ppk23*’s epistatic interactions mapped to *fruitless* (*fru*; human homolog *ZBTB24*), a transcription factor responsible for sex-specific neural phenotypes involved in courtship and pheromone detection [21] that co-localizes with *ppk23* on the leg and wing microchaete differentially between sexes, [18,19,22-24]. In addition to their co-localization in the peripheral nervous system, *ppk23* and *fru* have sex-specific co-localization patterns in the thoracic ganglion. This cluster of neurons is central to the “escape” response, allowing for ultra-fast processing of and response to flight-associated cues [25,26]. Males show more connections between *ppk23* and *fru* in the thoracic ganglion, and co-localization in neurons crossing the midline between the two sides of the anterior-most, pro-thoracic ganglion [18,19]. *fru* is also expressed in vMS2 motor neurons connecting the thoracic ganglion to the flight musculature, likely involved in courtship song generation and aggression behaviors [27,28]. The connection between *ppk23* and *fru*, and their co-localization in sensory and motor neurons affecting wing motion raise the possibility of a mechanistic connection between the sexual dimorphism observed in our study.

This hypothesized mechanic connection is bolstered by the identification of genes using PEGASUS_flies. One gene in particular, *doublesex* (*dsx*), is known to interact with *fru* and *ppk1* in patterning sex-specific neural networks for courtship. Another significant whole gene, *dissatisfaction* (*dsf*), a modifier of courtship behavior [27,29-31]. Other genes, such as *blue cheese (bchs), Ccn, CG13506, defective proboscis extension response 6 (dpr6), pollux (plx), sidekick (sdk), eiger (egr*) all have important implications in sex-specific behaviors that are known to affect fitness [22,32-34]. Further study of these genes may yield promising insights into the genetic basis of sex-specific behaviors.

References

1. Mummery-Widmer JL, Yamazaki M, Stoeger T, Novatchkova M, Bhalerao S, Chen D, et al. Genome-wide analysis of Notch signalling in Drosophila by transgenic RNAi. Nature. 2009;458(7241):987-U59. doi: 10.1038/nature07936. PubMed PMID: WOS:000265412900033.

2. Neumuller RA, Richter C, Fischer A, Novatchkova M, Neumuller KG, Knoblich JA. Genome-Wide Analysis of Self-Renewal in Drosophila Neural Stem Cells by Transgenic RNAi. Cell Stem Cell. 2011;8(5):580-93. doi: 10.1016/j.stem.2011.02.022. PubMed PMID: WOS:000290927600017.

3. Brown JB, Boley N, Eisman R, May GE, Stoiber MH, Duff MO, et al. Diversity and dynamics of the Drosophila transcriptome. Nature. 2014;512(7515):393-9. doi: 10.1038/nature12962. PubMed PMID: WOS:000340840600025.

4. Zheng Z, Zheng H, Yan W. Fank1 is a testis-specific gene encoding a nuclear protein exclusively expressed during the transition from the melotic to the haploid phase of spermatogenesis. Gene Expression Patterns. 2007;7(7):777-83. doi: 10.1016/j.modgep.2007.05.005. PubMed PMID: WOS:000249331000008.

5. DeGiorgio M, Lohmueller KE, Nielsen R. A Model-Based Approach for Identifying Signatures of Ancient Balancing Selection in Genetic Data. Plos Genetics. 2014;10(8). doi: 10.1371/journal.pgen.1004561. PubMed PMID: WOS:000341577800047.

6. Firth LC, Baker NE. Spitz from the retina regulates genes transcribed in the second mitotic wave, peripodial epithelium, glia and plasmatocytes of the Drosophila eye imaginal disc. Developmental Biology. 2007;307(2):521-38. doi: 10.1016/j.ydbio.2007.04.037. PubMed PMID: WOS:000248019100027.

7. Casas-Vila N, Bluhm A, Sayols S, Dinges N, Dejung M, Altenhein T, et al. The developmental proteome of Drosophila melanogaster. Genome Research. 2017;27(7):1273-85. doi: 10.1101/gr.213694.116. PubMed PMID: WOS:000404735500016.

8. Chintapalli VR, Wang J, Dow JA. Using FlyAtlas to identify better Drosophila melanogaster models of human disease. Nature genetics. 2007;39(6):715-20.

9. Harbison ST, Kumar S, Huang W, McCoy LJ, Smith KR, Mackay TFC. Genome-Wide Association Study of Circadian Behavior in Drosophila melanogaster. Behavior Genetics. 2019;49(1):60-82. doi: 10.1007/s10519-018-9932-0. PubMed PMID: WOS:000455331800005.

10. Dierick HA, Greenspan RJ. Molecular analysis of flies selected for aggressive behavior. Nature Genetics. 2006;38(9):1023-31. doi: 10.1038/ng1864. PubMed PMID: WOS:000240112100019.

11. Page RM, Munch A, Horn T, Kuhn PH, Colombo A, Reiner O, et al. Loss of PAFAH1B2 Reduces Amyloid-beta Generation by Promoting the Degradation of Amyloid Precursor Protein C-Terminal Fragments. Journal of Neuroscience. 2012;32(50):18204-14. doi: 10.1523/jneurosci.2681-12.2012. PubMed PMID: WOS:000312404700026.

12. Durham MF, Magwire MM, Stone EA, Leips J. Genome-wide analysis in Drosophila reveals age-specific effects of SNPs on fitness traits. Nature Communications. 2014;5. doi: 10.1038/ncomms5338. PubMed PMID: WOS:000340615500029.

13. Neely GG, Hess A, Costigan M, Keene AC, Goulas S, Langeslag M, et al. A Genome-wide Drosophila Screen for Heat Nociception Identifies alpha 2 delta 3 as an Evolutionarily Conserved Pain Gene. Cell. 2010;143(4):628-38. doi: 10.1016/j.cell.2010.09.047. PubMed PMID: WOS:000284149100019.

14. zur Lage P, Newton FG, Jarman AP. Survey of the Ciliary Motility Machinery of Drosophila Sperm and Ciliated Mechanosensory Neurons Reveals Unexpected Cell-Type Specific Variations: A Model for Motile Ciliopathies. Frontiers in Genetics. 2019;10. doi: 10.3389/fgene.2019.00024. PubMed PMID: WOS:000457405100001.

15. Brockington M, Yuva Y, Prandini P, Brown SC, Torelli S, Benson MA, et al. Mutations in the fukutin-related protein gene (FKRP) identify limb girdle muscular dystrophy 2I as a milder allelic variant of congenital muscular dystrophy MDC1C. Human Molecular Genetics. 2001;10(25):2851-9. doi: 10.1093/hmg/10.25.2851. PubMed PMID: WOS:000172868200001.

16. Inlow JK, Restifo LL. Molecular and comparative genetics of mental retardation. Genetics. 2004;166(2):835-81. doi: 10.1534/genetics.166.2.835. PubMed PMID: WOS:000220390600020.

17. Vannoy CH, Xiao W, Lu PJ, Xiao X, Lu QL. Efficacy of Gene Therapy Is Dependent on Disease Progression in Dystrophic Mice with Mutations in the FKRP Gene. Molecular Therapy-Methods & Clinical Development. 2017;5:31-42. doi: 10.1016/j.omtm.2017.02.002. PubMed PMID: WOS:000406299600004.

18. Lu BK, LaMora A, Sun YS, Welsh MJ, Ben-Shahar Y. ppk23-Dependent Chemosensory Functions Contribute to Courtship Behavior in Drosophila melanogaster. Plos Genetics. 2012;8(3). doi: 10.1371/journal.pgen.1002587. PubMed PMID: WOS:000302254800062.

19. Thistle R, Cameron P, Ghorayshi A, Dennison L, Scott K. Contact chemoreceptors mediate male-male repulsion and male-female attraction during Drosophila courtship. Cell. 2012;149(5):1140-51.

20. Gorczyca DA, Younger S, Meltzer S, Kim SE, Cheng L, Song W, et al. Identification of Ppk26, a DEG/ENaC Channel Functioning with Ppk1 in a Mutually Dependent Manner to Guide Locomotion Behavior in Drosophila. Cell Reports. 2014;9(4):1446-58. doi: 10.1016/j.celrep.2014.10.034. PubMed PMID: WOS:000345529600025.

21. Kimura KI, Ote M, Tazawa T, Yamamoto D. Fruitless specifies sexually dimorphic neural circuitry in the Drosophila brain. Nature. 2005;438(7065):229-33. doi: 10.1038/nature04229. PubMed PMID: WOS:000233133500050.

22. Pavlou HJ, Goodwin SF. Courtship behavior in Drosophila melanogaster: towards a 'courtship connectome'. Current Opinion in Neurobiology. 2013;23(1):76-83. doi: 10.1016/j.conb.2012.09.002. PubMed PMID: WOS:000314562900013.

23. Ben-Shahar Y. Sensory Functions for Degenerin/Epithelial Sodium Channels (DEG/ENaC). In: Friedmann T, Dunlap JC, Goodwin SF, editors. Advances in Genetics, Vol 76. Advances in Genetics. 762011. p. 1-26.

24. Gendron CM, Kuo TH, Harvanek ZM, Chung BY, Yew JY, Dierick HA, et al. Drosophila Life Span and Physiology Are Modulated by Sexual Perception and Reward. Science. 2014;343(6170):544-8. doi: 10.1126/science.1243339. PubMed PMID: WOS:000330343700048.

25. Strausfeld NJ. Brain and optic lobes. Encyclopedia of Insects: Elsevier; 2009. p. 121-30.

26. Lehmann FO, Bartussek J. Neural control and precision of flight muscle activation in Drosophila. Journal of Comparative Physiology a-Neuroethology Sensory Neural and Behavioral Physiology. 2017;203(1):1-14. doi: 10.1007/s00359-016-1133-9. PubMed PMID: WOS:000393670200001.

27. Yu JY, Kanai MI, Demir E, Jefferis G, Dickson BJ. Cellular Organization of the Neural Circuit that Drives Drosophila Courtship Behavior. Current Biology. 2010;20(18):1602-14. doi: 10.1016/j.cub.2010.08.025. PubMed PMID: WOS:000282385600020.

28. Ewing AW. NEUROMUSCULAR BASIS OF COURTSHIP SONG IN DROSOPHILA - ROLE OF THE DIRECT AND AXILLARY WING MUSCLES. Journal of Comparative Physiology. 1979;130(1):87-93. doi: 10.1007/bf02582977. PubMed PMID: WOS:A1979GS00900010.

29. Shirangi TR, Wong AM, Truman JW, Stern DL. Doublesex Regulates the Connectivity of a Neural Circuit Controlling Drosophila Male Courtship Song. Developmental Cell. 2016;37(6):533-44. doi: 10.1016/j.devcel.2016.05.012. PubMed PMID: WOS:000378204200008.

30. Rezaval C, Pavlou HJ, Dornan AJ, Chan YB, Kravitz EA, Goodwin SF. Neural Circuitry Underlying Drosophila Female Postmating Behavioral Responses. Current Biology. 2012;22(13):1155-65. doi: 10.1016/j.cub.2012.04.062. PubMed PMID: WOS:000306379600018.

31. Finley KD, Taylor BJ, Milstein M, McKeown M. dissatisfaction, a gene involved in sex-specific behavior and neural development of Drosophila melanogaster. Proceedings of the National Academy of Sciences. 1997;94(3):913-8.

32. dos Santos G, Schroeder AJ, Goodman JL, Strelets VB, Crosby MA, Thurmond J, et al. FlyBase: introduction of the Drosophila melanogaster Release 6 reference genome assembly and large-scale migration of genome annotations. Nucleic Acids Research. 2015;43(D1):D690-D7. doi: 10.1093/nar/gku1099. PubMed PMID: WOS:000350210400101.

33. Billeter JC, Villella A, Allendorfer JB, Dornan AJ, Richardson M, Gailey DA, et al. Isoform-specific control of male neuronal differentiation and behavior in Drosophila by the fruitless gene. Current Biology. 2006;16(11):1063-76. doi: 10.1016/j.cub.2006.04.039. PubMed PMID: WOS:000238245900019.

34. Finley KD, Taylor BJ, Milstein M, McKeown M. dissatisfaction, a gene involved in sex-specific behavior and neural development of Drosophila melanogaster. Proceedings of the National Academy of Sciences of the United States of America. 1997;94(3):913-8. doi: 10.1073/pnas.94.3.913. PubMed PMID: WOS:A1997WG23400026.
